# Different imaging techniques for the detection of pelvic lymph nodes metastasis from gynecological malignancies: a systematic review and meta-analysis

**DOI:** 10.18632/oncotarget.12959

**Published:** 2016-10-27

**Authors:** Yi Gong, Qingming Wang, Li Dong, Yiping Jia, Chengge Hua, Fanglin Mi, Chunjie Li

**Affiliations:** ^1^ Department of Obstetrics and Gynaecology, The First Affiliated Hospital of Nanchang University, Nanchang, Jiangxi, China; ^2^ Department of Hematology, The Second Affiliated Hospital of Nanchang University, Nanchang, Jiangxi, China; ^3^ Department of Cardiology, The Second Affiliated Hospital of Southwest Medical University, Lu Zhou, Sichuan, China; ^4^ Department of Ultrasound, No.4 West China Teaching Hospital, Sichuan University, Chengdu, China; ^5^ Department of Oral and Maxillofacial Surgery, Department of Evidence-based Dentistry, West China Hospital of Stomatology, State Key Laboratory of Oral Diseases, Sichuan University, Chengdu, China; ^6^ Department of Stomatology, Affiliated Hospital of North Sichuan Medical College, Nanchong, Sichuan, China; ^7^ Department of Head and Neck Oncology, Department of Evidence-based Dentistry, West China Hospital of Stomatology, State Key Laboratory of Oral Diseases, Sichuan University, Chengdu, China

**Keywords:** imaging technique, pelvic lymph node, metastasis, gynecological malignance, systematic review and meta-analysis

## Abstract

**Objective:**

This study aimed to evaluate the diagnostic performance of different imaging techniques and the corresponding diagnostic criteria for preoperative detection of pelvic lymph node metastasis from gynecological carcinomas.

**Methods:**

Six databases were systematically searched for retrieving eligible studies. Study inclusion, data extraction and risk of bias assessment were performed by 2 reviewers independently. STATA 14.0 was used to perform the meta-analysis. Results: Eighty eligible studies were collected. The pooled sensitivity, specificity, and area under curve (AUC) of CT, MRI and DWI were 47%, 93%, 0.7424; 50%, 95%, 0.8039 and 84%, 95%, 0.9523 respectively. As regards PET, PET-CT and US, the pooled sensitivity, specificity and AUC were 56%, 97%, 0.9592; 68%, 97%, 0.9363 and 71%, 99%, 0.9008 respectively. The summary receiver operating characteristic (SROC) curve indicated that the systematic diagnostic performances of PET, PET-CT, DWI were superior to other imaging modalities.

**Conclusions:**

The present work demonstrated that DWI, PET, PET-CT were the top-priority consideration of imaging modalities for detecting metastatic pelvic lymph node in gynecological carcinoma. DWI was recommended as the first choice for metastasis exclusion and all the other imaging techniques including CT and MRI were suitable for metastasis conformation. However, for the early stage lymph node malignancy, PET or PET-CT could represent a better choice. More studies exploring the diagnostic efficacy of detailed criteria are required in the future.

## INTRODUCTION

Gynecological carcinoma, including uterine cervix cancer, uterine corpus cancer, uterine endometrial cancer and ovarian cancer, is one of the leading causes of cancer death among women [1]. Pelvic lymph nodes metastasis is associated with poor clinical outcome in gynecological cancer patients [2]. In addition, the presence of metastatic lymph nodes strongly influences the option of treatment modalities including the necessity or extent of pelvic lymph node dissection [3, 4]. Therefore, it is of utmost importance to distinguish the normal pelvic lymph nodes from the metastatic ones before operation.

To assess the involvement of pelvic lymph nodes by gynecological carcinoma, several imaging techniques including magnetic resonance imaging (MRI), computed tomography (CT), positron emission tomography-computed tomography (PET-CT), were employed in preoperative lymph node inspection [5–7]. However, the accuracy of these imaging modalities is inconstant, as shown by different reports [8, 9]. On the other hand, criteria adopted in the evaluation of lymph node metastasis are inconsistent. Therefore, the current meta-analysis was performed to determine the diagnostic efficacy of imaging modalities employed in detection of pelvic lymph node metastasis from gynecological carcinomas and corresponding diagnostic criteria.

## RESULTS

### Results of the search

**Figure 1:**
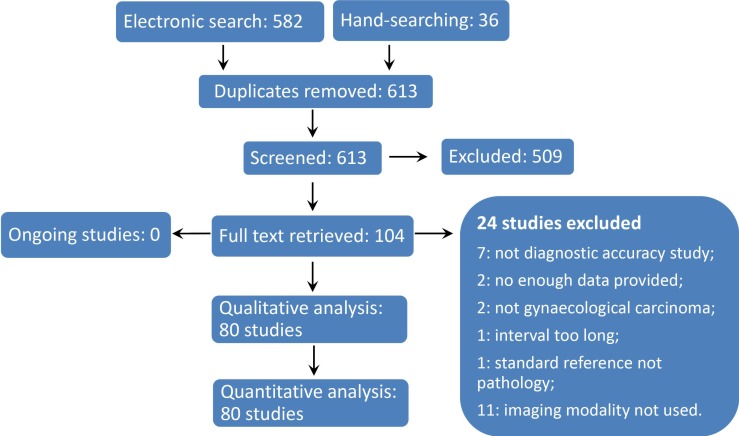
Flow Diagram of Study Inclusion

We retrieved 613 records by electronic search and hand-searching after removing duplicates. We identified 104 potential eligible studies and we obtained the full-text of these studies. Finally, 80 studies fulfilled the inclusion criteria were included [10–89] (Figure 1).

### Characteristics of included studies

The 80 included studies involved 5817 participants in the age range of 12-94 years. All the participants were diagnosed as gynecological cancers. The imaging modalities used in the included studies were MRI, diffusion weighted imaging (DWI), CT, PET, PET-CT and Ultrasonography (US). The analysis units used were lymph node, anatomical region, hemipelvic and patient. As regards the clinical significance, the analytical unit including anatomical regions, hemipelvic and patient were considered as region/patient unit in the subsequent analysis and we only chose region/patient as unit of analysis when multiple analytical units were eligible. As regards the study design, 33 were prospective and the others were retrospective. A detailed description of the characteristics of each included study is listed in Table 1.

**Table 1:** Characteristics of Included Studies

Study ID	Country/ Region	Study type	N	Age	Types of cancer	Targeted lymph nodes	Unit of analysis	Imaging
Antonsen 2013 [10]	Denmark	P	268*	65(29-94)	Endometrial cancer	PLN/PALN	Patient	PET-CT
Bandy 1985 [11]	USA	R	44	-	Carcinoma of cervix	PLN/PALN	Patient	CT
Boonya-ussadorn 2014 [12]	Thailand	R	33	53.7	Endometrial cancer	PLN	Patient/Region	PET-CT, CT/MRI
Cabrita 2008 [13]	Portugal	P	162	64.6(22-94)	Endometrial carcinoma	PLN	Patient	MRI
Camilien 1988 [14]	USA	R	51	-	Carcinoma of cervix	PLN/PALN	Patient	CT
Chao 2006 [15]	Taiwan	P	49	55(30-81)	Endometrial cancer	PLN/PALN	Region	PET, CT/MRI
Chen 2011-a [16]	China	P	61	46(25-60)	Uterine cervical cancer	PLN	Node	MRI, DWI
Chen 2011-b [17]	China	P	26	35(26-58)	Cervical carcinoma	PLN	Node	DWI
Chen 2016 [18]	China	R	33	50.3(28-69)	Endometrial cancer	PLN	Patient	MRI
Cheng 1999 [19]	Taiwan	P	104	50.6	Carcinoma of Cervix	PLN	Patient	TVS
Cheung 1998 [20]	Hong Kong	P	32*	45(21-64)	Cervical cancer	PLN	Patient	LS, CT
Choi 2006-a [21]	Korea	P	22	50(25-65)	Uterine cervical carcinoma	PLN/PALN	Patient/Region	MRI, PET-CT
Choi 2006-b [22]	Korea	R	55	48(18-65)	Uterine cervical cancer	PLN/PALN	Region/Node	MRI
Chou 2006 [23]	Taiwan	P	60	48(28-75)	Cervical cancer	PLN	Patient	PET
Chung 2007 [24]	Korea	R	119	50(28-81)	Cervical cancer	PLN/PALN	Patient/Region	MRI
Chung 2009 [25]	Korea	R	34	45.5(28-70)	Cervical cancer	PLN	Patient/Region	PET-CT
Chung 2010 [26]	Korea	R	83	47(24-80)	Cervical cancer	PLN/PALN	Patient	MRI, PET-CT
Crivellaro 2013 [27]	Italy	P	76	62.9(27-86)	Endometrial cancer	PLN/PALN	Patient/Region	PET-CT
Driscoll 2015 [28]	Ireland	R	47	48(22-86)	Uterine cervical cancer	PLN	Patient	PET-CT
Greco 1989 [29]	UK	R	46	-	Cervical carcinoma	PLN	Patient	MRI
Grumbine 1981 [30]	USA	R	24	-	Cervical cancer	PLN	Patient	CT
Han 2010 [31]	Korea	R	300*	-	Endometrial cancer	PLN	Patient	CT/MRI
Hawighorst 1998 [32]	Germany	P	33	55(20-68)	Uterine cervical cancer	PLN	Patient	MRI
Hawnaur 1994 [33]	UK	R	50*	18-55	Carcinoma of cervix	PLN	Patient/Node	MRI
Henrich 2007 [34]	Germany	P	39	61.5(38-87)	Ovarian cancer	PLN	Patient	TVS
Horowitz 2004 [35]	USA	P	19	66(54-90)	Uterine corpus cancer	PLN/PALN	Patient/Region	PET
Huang 2011 [36]	China	R	196	50.3(28-69)	Endometrial cancer	PLN	Patient	DWI
Husby 2015 [37]	Norway	P	102	-	Endometrial carcinoma	PLN	Patient	PET-CT
Inubashiri 2009 [38]	Korea	R	46	56(37-87)	Uterine corpus cancer	PLN	Patient/Region	PET, CT, MRI
Janus 1989 [39]	USA	P	22	25-66	Cervical carcinoma	PLN	Patient	CT, MRI
Keller 2004 [40]	Switzerland	P	13*	51	Cervical/Endometrial/Vulvar cancer	PLN	Patient	MRI
Kim 1990 [41]	Korea	P	30	48(31-70)	Uterine cervical carcinoma	PLN	Region	CT, MRI
Kim 1993 [42]	Korea	R	99	48(27-71)	Uterine cervical carcinoma	PLN	Hemi-pelvic	CT, MRI
Kim 1994 [43]	Korea	R	136	49(26-71)	Uterine cervical cancer	PLN	Hemi-pelvic	MRI
Kim 2008 [44]	Korea	R	125	48	Uterine cervical cancer	PLN	Patient/Region	DWI
Kim 2011 [45]	Korea	R	143	48(24-73)	Uterine cervical cancer	PLN	Node	MRI, DWI
Kitajima 2008 [46]	Japan	P	40	56(43-77)	Endometrial cancer	PLN/PALN	Patient/Node	PET-CT
Kitajima 2013 [47]	Japan	R	30	62.4(30-88)	Endometrial cancer	PLN	Patient	PET-MRI, PET-CT,MRI
Klar 2010 [48]	Germany	P	13	-	Uterine corpus cancer	PLN/PALN	Patient	PET
Klerkx 2012 [49]	Netherlands	P	68	-	Early stage cervical cancer	PLN	Region	MRI, DWI
Koplay 2014 [50]	Turkey	R	58	58(42-78)	Endometrial cancer	PLN	Patient	DWI
Li 2011-a [51]	China	R	42	49(39-67)	Cervical/Endometrial cancer	Internal iliac	Patient	TVS
Li 2011-b [52]	China	R	62	51(35-65)	Cervical/Endometrial cancer	Obturator	Patient	TVS
Liao 2008 [53]	China	P	53	45 (25-59)	Cervical carcinoma	PLN	Node	MRI, DWI
Liu 2011 [54]	China	R	42	45.3 (30-62)	Uterine cervical cancer	PLN	Node	MRI, DWI
Lv 2015 [55]	China	R	87	55(45-73)	Cervical cancer	PLN	Patient/Node	PET-CT, MRI
Ma 2009 [56]	China	R	275	21-70	Uterine cervical carcinoma	PLN	Hemi-pelvic	CT
Matsukuma 1989 [57]	Japan	R	70	-	Carcinoma of cervix	PLN/PALN	Patient/Region	CT
Nakamura 2010 [58]	Japan	R	44	60.5(47-87)	Endometrial cancer	PLN	Patient	PET-CT
Nakamura 2011 [59]	Japan	R	106	59.32(34-87)	Endometrial cancer	PLN	Patient	PET-CT
Nogami 2014 [60]	Japan	R	123	-	Cervical/Endometrial cance	PLN	Patient	PET-CT
Ozalp 1999 [61]	Turkey	P	37	54.3(12-75)	Ovarian/endometrial cancer	PLN/PALN	Patient	CT
Park 2008 [62]	Korea	R	53	52(27-68)	Uterine corpus cancer	PLN/PALN	Patient/Region	PET-CT, MRI
Picchio 2010 [63]	Italy	R	32*	61(35-79)	Endometrial cancer	PLN	Patient	PET-CT
Rechichi 2013 [64]	Italy	R	40	56(45-69)	Endometrial cancer	PLN	Region	DWI, MRI
Reinhardt 2001 [65]	Germany	P	35	49(26-78)	Cervical cancer	PLN, PALN	Patient/Region	MRI, PET
Rizzo 2014 [66]	Italy	P	217	46.2	Cervical cancer	PLN	Region	MRI
Sawicki 2003 [67]	Poland	P	90	63.3(32-86)	Endometrial cancer	PLN	Patient	TVS
Signorelli 2009 [68]	Italy	P	37	61(59-78)	Endometrial cancer	PLN	Patient/Region	PET-CT
Signorelli 2013 [69]	Italy	P	68	42(16-91)	Ovarian cancer	PLN/PALN	Patient/Region	PET-CT
Sironi 2006 [70]	Italy	P	47	(29-71)	Cervical cancer	PLN	Patient	PET-CT
Subak 1995 [71]	USA	R	79*	53.3(29-82)	Cervical cancer	PLN	Patient	CT, MRI
Sufian 2014 [72]	Pakistan	R	48	55(20-79)	Carcinoma endometrium, Cervix or ovary cancer	PLN	Node	MRI
Suga 2011 [73]	Japan	R	40*	56(27-81)	Endometrial cancer	PLN/PALN	Patient/Region	PET/PET-CT
Sugiyama 1995 [74]	Japan	R	95	-	Ovarian cancer	PLN/PALN	Patient	CT
Suzuki 2007 [75]	Japan	P	30	55.4(27-73)	Endometrial cancer	PLN/PALN	Patient	PET, CT/MRI
Teng 2015 [76]	China	R	167	57.9(34-80)	Endometrial carcinoma	PLN	Patient	MRI
Unger 2005 [77]	USA	R	13	40.8(33.6-48)	Cervical cancer	PLN	Patient	PET, CT
Van Engelshoven 1984 [78]	Netherlands	R	56*	-	Cervical cancer	PLN	Patient	CT
Vas 1985 [79]	USA	R	59*	28-91	Carcinoma of cervix	PLN/PALN	Patient	CT
Vijaykumar 1995 [80]	India	R	75	-	Cervical carcinoma	PLN	Patient	TAS
Walsh 1981 [81]	USA	P	77*	56(30-90)	Carcinoma of cervix	PLN	Patient	CT
Wang 2015 [82]	China	R	104	44.3(21-67)	Ovarian malignant tumor	PLN	Patient	PET-CT
Wright 2005 [83]	USA	R	59	43	Cervical carcinoma	PLN/PALN	Patient/Node	PET
Xue 2008 [84]	China	R	24	37.9	Carcinoma of cervix	PLN	Node	DWI
Yang 1999 [85]	Hong Kong	P	31	45(21-64)	Uterine cervical cancer	PLN	Region	LS
Yang 2000 [86]	Hon Kong	P	43	46(21-79)	Uterine cervical cancer	PLN	Hemi-pelvic/Node	CT, MRI
Yeh 2002 [87]	Taiwan	P	42	-	Uterine cervical cancer	PLN/PALN	Patient	PET
Yoo 2009 [88]	Korea	R	99	49 (29-78)	Uterine cancer	PLN	Node	MRI
Zhang 2014 [89]	China	R	125	42.51 (25-72)	Cervical cancer	PLN	Patient	MRI/DWI

Abbreviations: P, prospective; R, retrospective; MRI, magnetic resonance imaging; PET, positron emission tomography; CT, computed tomography; DWI, diffusion-weighted imaging; PET-CT, positron emission tomographyplus computed tomography; PLN, pelvic lymph nodes; PALN, para-aortic lymph nodes; TVS, Trans-vaginal ultrasound; LS, Laparoscopic sonography; TAS, Transabdominal Ultrasound; ROI, region of interest; CT/MRI, some patients got CT and others got MRI, mixed data reported with no separate data; MRI/DWI, some patients got DWI and others got MRI, mixed data reported with no separate data; PET/PET-CT, some patients got PET and others got PET-CT, mixed data reported with no separate data

* only part of patients were included in present study.

### Quality of included studies

**Figure 2:**
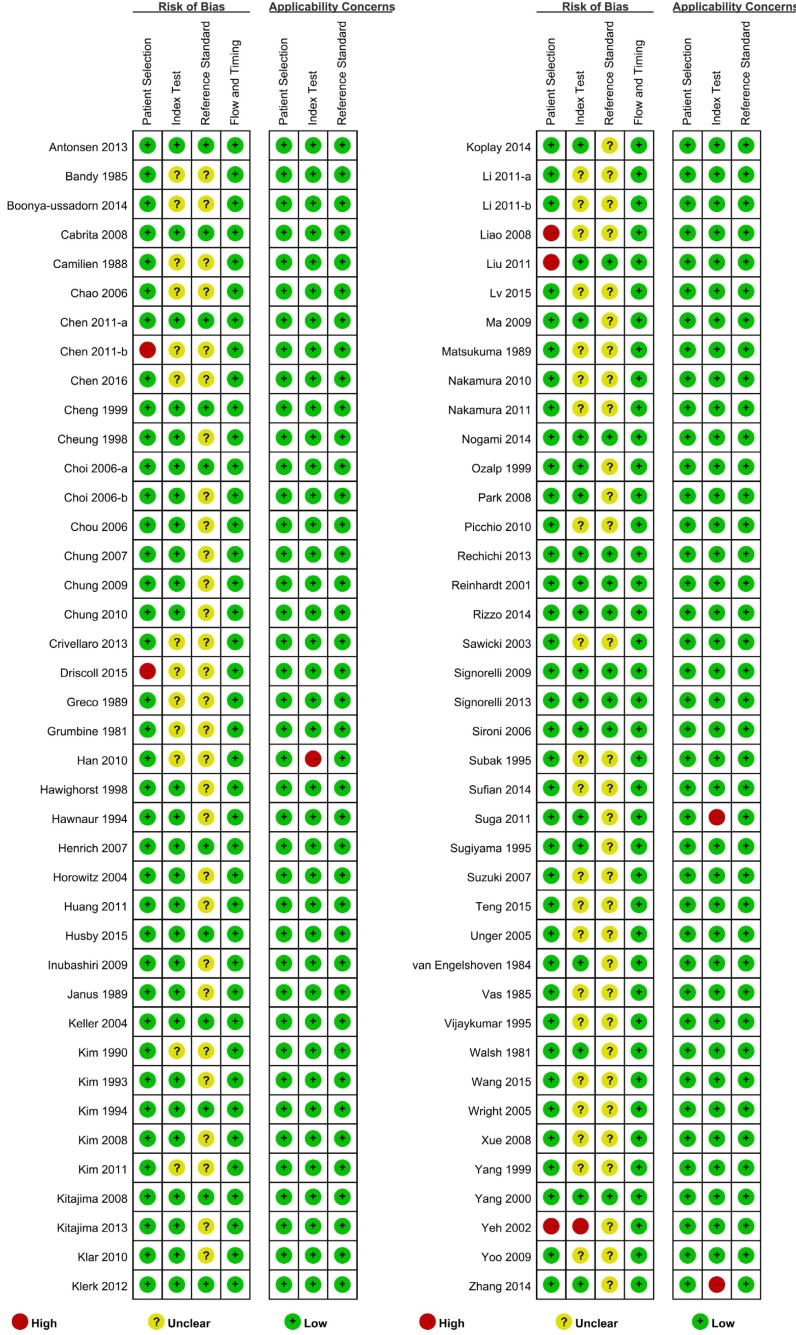
Summary Qualities of Included Studies

The risk of bias assessment revealed that 19 studies had low risk of bias and 56 had unclear risk of bias; the remaining five had high risk of bias. Driscoll 2015[28] included only MRI negative patients who underwent PET-CT examination, which was considered as high risk of inappropriate exclusion. For Yeh 2002 [87], the authors used the criteria that only MRI negative patients could be included but they also mentioned that radiologists were not blinded, thus inducing a high risk of bias. For Chen 2011-b [17], Liao 2008 [53] and Liu 2011 [54], they had improper exclusion which were considered as high risk of bias. As regards the applicability assessment, 77 studies had low applicability concerns, three with high applicability concerns, because the three studies used two imaging modalities without reporting the diagnostic efficacy of each imaging modality independently (Figure 2).

### Diagnostic efficacy of each imaging modality

MRI. A total of 36 studies assessed the diagnostic efficacy of MRI to identify pelvic metastatic lymph nodes caused by gynecological cancers. Meta-regression was performed to identify any potential clinical and methodological heterogeneity that could alter the outcome. Meta-regression suggested that the use of DWI might have a significant influence on the outcome including summarized diagnostic efficacy (*P* = 0.01) and sensitivity (SEN) (*P* = 0.00) (Table 2, Appendix Table 2 and 3). So we made subgroup analysis instead of combining all MRI studies together.

**Table 2:** Meta-regression of MRI on Diagnostic Efficacy

Parameter	I-squared (95%CI)	LRT Chi	*P* value
Publication year	0.00 [0.00 - 100.00]	0.96	0.62
Types of studies	0.00 [0.00 - 100.00]	0.59	0.75
Slide thickness	0.00 [0.00 - 100.00]	0.96	0.62
Intersection gap	0.00 [0.00 - 100.00]	0.53	0.77
Enhanced	12.01[0.00 - 100.00]	2.27	0.32
Combined with DWI	76.35[48.28–100.00]	8.46	0.01
Blind of radiologist	0.00 [0.00 - 100.00]	1.15	0.56
Blind of pathologist	0.00 [0.00 - 100.00]	1.96	0.37

**Table 3:** Summary of Meta-analysis (Region/Patient as Unit of Analysis)

	N^#^	SEN	SPE	+LR	-LR	DOR	AUC	Q*
MRI	27	0.50[0.42,0.57]	0.95[0.92,0.97]	9.20[6.12,13.81]	0.53[0.46,0.62]	17.23[11.10,26.76]	0.8039(0.0435)	0.7427(0.0352)
DWI	6	0.84[0.54,0.96]	0.95[0.88,0.98]	15.51[5.93,40.57]	0.17[0.05,0.60]	91.80[12.86,655.54]	0.9523(0.0243)	0.9062(0.0506)
CT	18	0.47[0.39,0.56]	0.93[0.89,0.96]	7.21[4.44,11.70]	0.56[0.48,0.66]	12.81[7.59,21.61]	0.7424(0.0672)	0.6928(0.0523)
PET	10	0.56[0.34,0.77]	0.97[0.95-0.98]	19.44[8.22,45.97]	0.45[0.26,0.76]	43.23[11.65,160.41]	0.9592(0.0266)	0.9201(0.0605)
PET-CT	20	0.68[0.56,0.78]	0.97[0.94,0.98]	20.45[11.96,34.97]	0.34[0.24,0.47]	60.99[29.66,125.39]	0.9363(0.0336)	0.8729(0.0401)
Ultrasound	8	0.71[0.44,0.89]	0.99[0.83,1.00]	45.41[3.63,566.62]	0.29[0.13,0.66]	155.82[8.87,2737.46]	0.9008(0.0859)	0.8354(0.0828)
MRI or DWI	1	0.72	0.86					
CT or MRI	4	0.51(0.38-0.63)	0.96(0.95-0.98)	11.83(3.38-41.37)	0.53(0.41-0.67)	24.03(5.75-100.42)	0.5545(0.1625)	0.5409(0.1224)

^#^Number of included studies.

**Figure 3:**
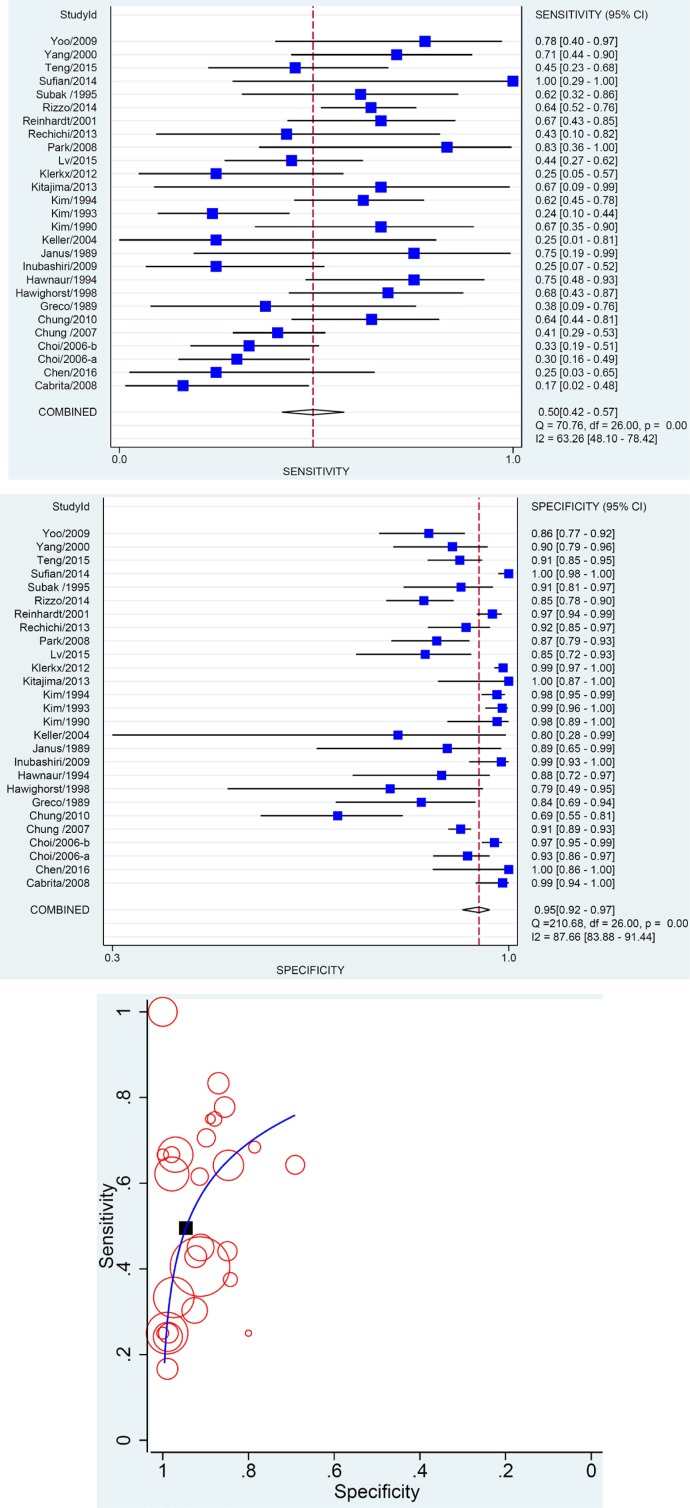
Results of Meta-analysis Assessing Diagnostic Efficacy of MR **A**. Pooled SEN; **B**. Pooled SPE; **C**. SORC curve. Every circle on the SROC curve represents the coordinate of SEN and SPE in a single study. And the black square represents the summary point where Q* locates.

A number of 31 studies evaluated traditional MRI diagnostic efficacy, with 27 studies reporting the results using region/patient as analytical unit, 8 using lymph node as unit and 4 using both. The meta-analysis based on region/patient as unit of analysis indicated a pooled SEN of 0.50, specificity (SPE) of 0.95, area under curve (AUC) of 0.8039 and a Q* of 0.7427 (Figure 3, Table 3). When node was identified as analytical unit, the pooled SEN was 0.52 but SPE reached 0.95 (Appendix Table 4).

**Table 4:** Subgroup Analysis of US

	N^#^	SEN	SPE	AUC	Q*
Laparoscopic US	2	0.92[0.75,0.99]	1.00[0.94,1.00]		
TransvaginalUS	5	0.55[0.44,0.66]	0.82[0.76,0.86]	0.8759(0.1317)	0.8063(0.1317)
TransabdominalUS	1	0.67[0.38,0.88]	0.78[0.66,0.88]		

^#^Number of included studies.

Only 11 studies assessed DWI and 6 studies used region/patient as analytical unit and 5 used node as analytical unit. When region/patient was considered as analytical unit, the pooled SEN was 0.84 and SPE was 0.95. In addition, the SROC curve suggested an AUC of 0.9523 and a Q* of 0.9062 (Table 3, Appendix Figure 1). The node-based results are shown in Appendix Table 4.

To confirm the results of meta-regression, we further conducted pair-wise comparison between MRI and DWI on the pooled results. The results showed that as regards SEN, DWI had a significant advantage over MRI (*P* = 0.0006), while as regards SPE, no difference between the two treatment modalities was detected (*P* = 1.00). Finally, as regards AUC and Q*, DWI had a significant advantage over MRI (*P* = 0.003 and 0.007, respectively). This result also confirmed the meta-regression.

In the meta-regression analysis for slide thickness and intersection gap, some including data are unclear. This may influence the result of meta-regression. To eliminate the effect from unclear data, we removed the related studies and conducted pair-wise comparison between ≤4mm and >4mm subgroup with slide thickness data and ≤1mm and >1mm subgroup with intersection gap data respectively.

The results showed that as regards SEN, AUC and Q*, both slide thickness and intersection gap had no obvious effect on diagnostic performance of MRI (*P* ≥ 0.05). However, as regards SPE, no difference between the two subgroups based on slide thickness was detected (*P* = 0.34) while significant difference was found between the subgroups based on intersection gap (*P* < 0.0001). This result was slightly inconsistent with the result of meta-regression on SPE for intersection gap.

#### CT

A number of 18 studies investigated the diagnostic efficacy of CT. All of them used region/patient as unit of analysis. Pooled SEN was 0.47 with 95% CI [0.39, 0.56] and SPE was 0.93 [0.89, 0.96]. SROC curve indicated an AUC of 0.7424 and Q* of 0.6928 (Table 3, Appendix Figure 2). One study reported the node-based results and is shown in Appendix Table 4.

#### PET and PET-CT

Among the included studies, 30 of them reported the diagnostic efficacy of PET and PET-CT. Meta-regression showed that none of the covariates could impact the result (Appendix Table 5-7). As we considered the natural difference of PET and PET-CT, we also made subgroup analysis considering each of them separately. PET subgroup analysis showed a pooled SEN of 0.56 and SPE of 0.97, while PET-CT showed a pooled SEN and SPE of 0.68 and 0.97, respectively (Table 3, Appendix Figure 3 and 4, node-based results in Appendix Table 4).

**Figure 4:**
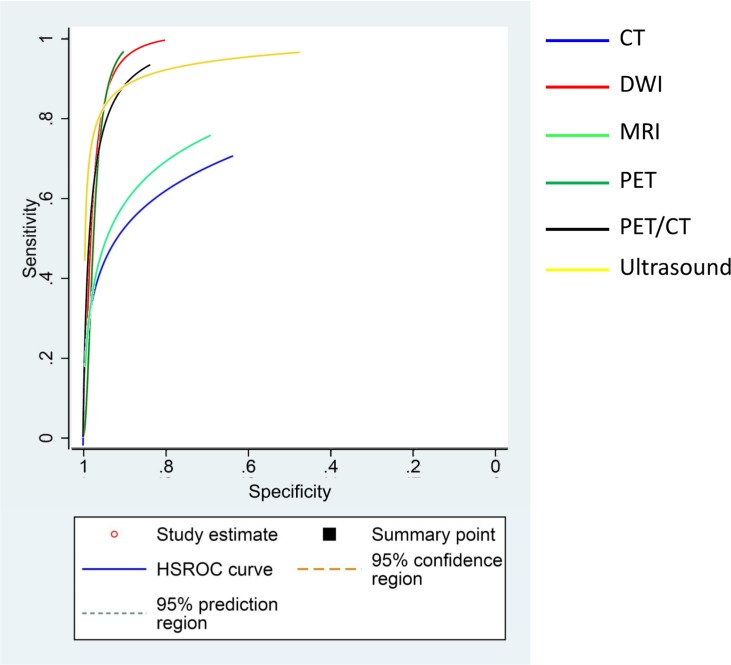
Combination of SROC curves from all kinds of imagings

We also conducted pair-wise analysis between PET and PET-CT. We used z test to compare related diagnostic parameters and confirm the results of meta-regression. No difference was found among SEN, SPE, AUC and Q* (*P* > 0.05).

Nine studies included only patients with an early stage gynecological carcinoma (FIGO stage I/II) (the FIGO stages are: stage 0: carcinoma in situ (common in cervical, vaginal, and vulval cancer); stage I: confined to the organ of origin; stage II: invasion of surrounding organs or tissue; stage III: spread to distant nodes or tissue within the pelvis; stage IV: distant metastasis(es)). The pooled results showed PET or PET-CT had an AUC and Q* of 0.9152 and 0.8497, respectively.

#### US

We retrieved 8 studies focusing on the diagnostic efficacy of US. Meta-analysis indicated a SEN of 0.71 and a SPE of 0.99 (Table 3, Appendix Figure 5, node-based results in Appendix Table 4). A subgroup analysis based on different US techniques was also performed, indicating that laparoscopic US had the highest diagnostic efficacy (Table 4).

### Diagnostic efficacy among different imaging modalities

To evaluate the diagnostic efficacy of different imaging techniques, we compared SEN, SPE and AUC of the imaging techniques considered. The results showed that DWI had the highest SEN, followed by US and PET-CT. As regards SPE, all the imaging techniques had a SPE >0.90, with US showing the highest SPE (Table 3). We also combined the SROC curve of all the imaging techniques, and the results showed that MRI and CT had a lower diagnostic efficacy compared to DWI, PET, PET-CT and US (Figure 4). The comparison between AUC and Q* among all the imaging techniques also indicated that DWI, PET, PET-CT and US had higher AUC and Q* values compared to CT or MRI (Table 2).

### Diagnostic efficacy of different diagnostic criteria

To detect the diagnostic efficacy of each diagnostic criterion, we summarized and synthesized all the reported criteria used in MRI, DWI, CT, PET, PET-CT studies. The diagnostic efficacy of each criterion used in various imaging techniques was listed in Table 5.

**Table 5:** Diagnostic Efficacy of Different Criteria

Criteria		No	Sensitivity	Specificity	AUC (SE)
**1. MRI**					
Short Axis	>4mm	2	0.78[0.69,0.86]	0.89[0.85,0.92]	
	>5mm	2	0.58[0.43,0.72]	0.72[0.69,0.75]	
	>6mm	2	0.50[0.35,0.65]	0.85[0.83,0.88]	
	>7mm	2	0.46[0.31,0.61]	0.91[0.89,0.93]	
	>8mm	5	0.60[0.53,0.68]	0.91[0.89,0.93]	0.8071(0.0674)
	>9mm	2	0.33[0.20,0.48]	0.98[0.96,0.99]	
	>10mm	18	0.47[0.42,0.51]	0.86[0.84,0.87]	0.7164(0.0566)
	>10mm and/or central necrosis	1	0.45[0.23,0.68]	0.91[0.85,0.95]	
	>12mm	1	0.35[0.20,0.53]	1.00[0.98.1.00]	
	>14mm	1	0.24[0.12,0.41]	1.00[0.98,1.00]	
	>16mm	1	0.05[0.01,0.18]	1.00[0.98,1.00]	
Long Axis	>8mm	2	0.82[0.72,0.89]	0.47[0.44,0.50]	
	>9mm	1	0.93[0.82,0.99]	0.66[0.58,0.74]	
	>10mm	4	0.69[0.56,0.79]	0.84[0.63,0.94]	0.7832(0.0497)
	>10mm and central necrosis	1	0.18[0.07,0.33]	1.00[1.00,1.00]	
	>12mm	3	0.52[0.39,0.64]	0.87[0.85,0.90]	0.8177(0.1129)
	>14mm	3	0.45[0.32,0.57]	0.91[0.89,0.93]	0.8133(0.1636)
	>16mm	2	0.20[0.10,0.34]	0.96[0.94,0.98]	
	>18mm	1	0.16[0.06,0.32]	1.00[0.98,1.00]	
	>20mm	1	0.14[0.05,0.29]	1.00[0.98,1.00]	
Ratio (short:long)	>0.5	1	0.83[0.52,0.98]	0.45[0.40,0.50]	
	>0.6	1	0.50[0.21,0.79]	0.73[0.68,0.77]	
	>0.7	2	0.64[0.51,0.76]	0.73[0.69,0.77]	
	>0.8	3	0.51[0.40-0.62]	0.87[0.84,0.90]	0.6772(0.0684)
	>0.9	1	0.08[0.00,0.38]	0.96[0.94,0.98]	
	≥1.3	1	0.47[0.23,0.72]	0.86[0.75,0.94]	
Miscellaneous	Extracapsular spread	1	0.15[0.08,0.26]	1.00[0.96,1.00]	
	Lobulated	2	0.57[0.34,0.78]	0.83[0.79,0.86]	
	Spiculated	2	0.20[0.04,0.48]	0.99[0.97,0.99]	
	Indistinct	1	0.00[0.00,0.26]	0.98[0.96,0.99]	
	Necrosis	1	0.21[0.12,0.33]	0.94[0.87,0.98]	
	4-point grading system	2	0.38[0.25,0.53]	0.93[0.87,0.97]	
**2. DWI**					
Short Axis	>10mm	2	0.77[0.61,0.89]	0.92[0.87,0.95]	
Mean ADC	<0.0005	1	0.25[0.05,0.57]	0.93[0.90,0.95]	
	<0.00055	1	0.25[0.05,0.57]	0.85[0.81,0.88]	
	<0.0006	1	0.33[0.10,0.65]	0.76[0.71,0.80]	
	<0.00065	1	0.33[0.10,0.65]	0.74[0.69,0.78]	
	<0.0007	1	0.75[0.43,0.95]	0.51[0.46,0.56]	
	<0.0008	1	0.92[0.62,1.00]	0.27[0.23,0.32]	
	<0.0009	4	0.86[0.79,0.92]	0.79[0.77,0.82]	0.8606(0.1267)
	<0.00109	2	0.92[0.82,0.98]	0.83[0.78,0.88]	
	<0.00115	3	0.79[0.71,0.86]	0.82[0.76,0.87]	
Mean eADC	>0.335	1	0.71[0.57,0.83]	0.73[0.60,0.84]	
Minimum ADC	<0.000798	1	1.00[0.59,1.00]	0.99[0.95,1.00]	
	<0.000807	1	1.00[0.59,1.00]	0.98[0.93,1.00]	
	<0.00086	2	0.89[0.82,0.94]	0.74[0.70,0.77]	
	≤0.634	1	0.93[0.82,0.99]	0.91[0.85,0.95]	
Max ADC	<0.00097	1	0.79[0.67,0.87]	0.77[0.73,0.80]	
	<0.00183	1	0.92[0.80,0.98]	0.75[0.62,0.85]	
Relative ADC	<0.00028	1	0.80[0.69,0.89]	0.72[0.62,0.81]	
	<0.00072	1	0.85[0.71,0.94]	0.92[0.86,0.96]	
**3. CT**					
Short Axis	>8mm	1	0.65[0.38,0.86]	0.81[0.66,0.91]	
	>10mm	7	0.36[0.29,0.44]	0.93[0.92,0.94]	0.6120(0.0905)
Long Axis	>9mm	1	0.71[0.44,0.90]	0.86[0.71,0.95]	
	>10mm	2	0.63[0.52,0.73]	0.94[0.91,0.96]	
	>10mm and central necrosis	1	0.27[0.12,0.48]	1.00[1.00,1.00]	
	>14mm	5	0.41[0.32,0.50]	0.98[0.96,0.99]	0.8331(0.0968)
	>20mm	1	0.75[0.43,0.95]	0.91[0.75,0.98]	
Ratio (long:short)	≥1.3	1	0.41[0.18,0.67]	0.86[0.71,0.95]	
Miscellaneous	4-point grading system	1	0.31[0.11,0.59]	0.99[0.93,1.00]	
**4. PET**					
FDG uptake	Increased	5	0.57[0.42,0.71]	0.97[0.95,0.99]	0.9370(0.1071)
	close to primary tumor	1	1.00[0.16,1.00]	1.00[0.72,1.00]	
Miscellaneous	4-point grading system	1	0.31[0.11,0.59]	0.96[0.89,0.99]	
	5-point grading system	1	0.60[0.15,0.95]	0.99[0.92,1.00]	
**5. PET-CT**					
FDG uptake	Increased	13	0.54[0.46,0.61]	0.98[0.97,0.98]	0.7498(0.1871)
SUV	>11.0	1	0.73[0.54,0.87]	0.93[0.88,0.96]	
	>2 and short >10mm	1	0.91[0.71,0.99]	0.95[0.88,0.99]	
Max SUV	>3.0	1	0.71[0.29,0.96]	0.95[0.82,0.99]	
	>8.1	1	0.83[0.36,1.00]	0.91[0.84,0.96]	
	>=2.5 and short axis>5mm	1	0.69[0.39,0.91]	0.97[0.91,0.99]	
Miscellaneous	5-point grading system	2	0.79[0.67,0.88]	0.92[0.87,0.96]	

## DISCUSSION

Gynecological malignancy seriously threatens women health life [90]. Involvement of pelvic lymph nodes is one of the most important poor prognostic factors in gynecological malignancy [91–93]. Some studies demonstrated that 10-30% of women with stage I or II gynecological malignancy had pelvic lymph node metastasis and the probability of lymph node metastasis is even higher when this malignancy reach the stage III and IV, thus significantly impairing the prognosis [94–96]. Preoperative detection of pelvic lymph node metastasis is an important issue for gynecological surgeons, who can modulate the surgical plan and subsequent treatment according to the detection results. However, the achievement of a satisfactory detection is not a simple task. In clinical practice, a number of imaging modalities have been used to evaluate the lymph node status before surgery, including CT, MRI, PET, PET-CT and US [11, 13, 15, 21, 34]. Nevertheless, diagnostic efficacy of these techniques were reported inconsistently [21, 62, 86], thus, no unanimous conclusion could be drawn. On the other hand, no study assessed simultaneously all these imaging techniques, thus no study could provide information on which imaging technique had highest diagnostic efficacy. What's more, the large numbers of relevant papers available confuse the clinicians as they all report different diagnostic criteria. Therefore, we performed this systematic review and meta-analysis to provide clinical evidence for a better selection of preoperative imaging modalities and relevant criteria for the detection of metastatic pelvic lymph node. Furthermore, our study could help clinician to select the best surgical gynecological carcinoma procedure. As far as we know, this is the first meta-analysis systematically evaluating and comparing the diagnostic efficacy of almost all the relevant imaging approaches currently used in pelvic lymph node staging of gynecological malignancy. Besides, this systematic review is also the first one discussing the diagnostic efficacy of relevant criteria for a better evaluation of pelvic lymph node metastasis in gynecological carcinoma.

In this systematic review and meta-analysis, 80 eligible studies were recruited; six imaging modalities were used in these studies including CT, MRI, DWI, PET, PET-CT and US. The results of our study illustrated that the pooled SEN of these imaging techniques for detecting metastatic pelvic lymph nodes were 47% (CT), 50% (MRI), 84% (DWI), 56% (PET), 68% (PET-CT), 71% (US), while the polled SPE ranged from 93% to 99% with limited variation, with the highest SPE achieved by US (99%). The AUC values that represent the systematic diagnostic efficacy were 0.7424 (CT), 0.8039 (MRI), 0.9523 (DWI), 0.9592 (PET), 0.9363 (PET-CT), 0.9008 (US). Scheidler and colleagues [97] reported in 1997 CT and MRI diagnostic performance in the detection of metastatic pelvic lymph node in cervical cancer with a Q* value of 0.78 and 0.87, respectively, which are higher than our results. These results have caught our attention to explore why CT and MRI diagnostic performance several decades ago is superior to our results including studies with more advanced equipment. To clarify this issue, we calculated the SEN and SPE using the data provided by Scheidler's, and we obtained a SEN and SPE of 36% and 94% for CT, 57% and 96% for MRI, respectively, which are close to our result. In Scheidler's study, only 11 CT studies and 9 MRI studies were included, whereas 18 CT studies and 27 MRI studies were included in our study. The higher Q* value might derived from a relatively smaller number of included study and their heterogeneity. As regards DWI, Shen *et al* [98] evaluated the diagnostic performance of DWI in cervical cancer metastasis with a SEN, SPE and AUC of 86%, 84% and 0.9384, respectively. The pooled SEN and AUC are similar to our results, while the SPE of Shen is less than the SPE obtained in the present work. The predominant diagnostic performance of DWI was also confirmed by the result of Meta-regression in our study. In Shen's work, all the included studies were from 2 Asian countries (China and Korea), but in our current meta-analysis, we included studies from both Asia and Europe (China, Korea, Netherlands, Turkey and Italy), being thus more representative. The result suggests that DWI provided a better performance in distinguishing metastatic lymph node from benign lymph node. In addition, Chang *et al* [99] and Bollineni *et al* [9] conducted similar systematic reviews exploring diagnostic efficacy of PET and PET-CT in detection of pelvic lymph node metastasis in endometrial cancer, in which pooled SEN, SPE and AUC ranged from 63% to 72%, 94% to 94.7% and 0.94 to 0.953, respectively. Only 7 and 13 studies were included in Chang's and Bollineni's meta-analysis, respectively. Our results were consistent with Chang's and Bollineni's study although we included more studies. However, none of the above mentioned systematic reviews have evaluated all these imaging techniques simultaneously; thus, they could not evaluate which imaging technique has the highest diagnostic efficacy in the detection of pelvic lymph node metastasis from gynecological malignancies. That is exactly what we obtained in our systematic review. Based on the evidence provided by our current study, we suggest clinicians to proceed as follows. First, since DWI had a highest SEN, it represents the best technique for excluding pelvic lymph node metastasis in a patient with gynecological carcinoma. Second, all the imaging techniques we evaluated had a SPE higher than 93%, being suitable for confirming the diagnosis. Third, PET, DWI, PET-CT or US had a higher diagnostic efficacy than CT or MRI, thus they can be chosen as pre-operative scanning.

PET, PET-CT, DWI and US diagnostic efficiency found in the present study were clearly higher than conventional imaging modalities such as MRI or CT. This might be mainly due to the different imaging principles among these modalities. PET or PET-CT is a functional imaging, which relies on the increased fluoro-2-deoxy-D-glucose (FDG) uptake of malignant tissue [100]. This metabolism change usually precedes morphological changes, therefore, PET or PET-CT can detect lymph node malignant transformation at a relative early stage compared with MRI or CT, which mainly identify malignant transformation based on morphological changes [65]. In our study, data of 9 recruited studies using PET [23, 65, 77, 83] or PET-CT [25, 26, 28, 60, 70] in an early stage gynecological malignancies were combined, 1 study [28] even restricted inclusion criteria with negative MRI findings of metastatic pelvic lymph nodes. Pooled data demonstrated that the AUC and Q* value were 0.9152 and 0.8497, respectively, suggesting that PET or PET-CT were more suitable to conventional modalities such as CT (AUC, 0.7424; Q*, 0.6928) or MRI (AUC, 0.8039; Q*, 0.7427) in distinguishing metastatic pelvic lymph nodes in early stage patients.

Compared with radiological modalities, the US, especially transvaginal US, is safer, cost-effective and widely available. In clinical practice, laparoscopic, transvaginal and transabdominal US are frequently used in detection of metastatic lymph node. Among all kinds of US, laparoscopic US (number of studies: 2) had the highest SEN and SPE, followed by transvaginal US (number of studies: 5) with an AUC value of 0.8759. In the 5 transvaginal US studies, 2 of them restricted study objects to internal iliac and obturator lymph nodes which are anatomically close to vagina [52, 101]. The diagnostic accuracies were significantly higher in these 2 studies (0.92-0.95) than in the other 3 studies (0.46-0.89). However, the SPE in 4 of these 5 studies was high (94%-100%) [34, 52, 67, 101]. The results suggested that the detection of metastatic pelvic lymph nodes with transvaginal US might result in misdiagnosis, but it might be a good technique to confirm metastatic lymph node.

Since the diagnostic performances achieved by PET, PET-CT or DWI are similar and DWI is less expensive and available, we recommended DWI as the first option for the detection of pelvic lymph node in gynecological carcinoma. Nevertheless, PET or PET-CT might represent the better choice for the detection of micrometastatic lymph node in early stage carcinoma. Additionally, transvaginal US could be considered as a safe and economic technique for diagnosis confirmation of the internal iliac and obturator lymph nodes metastasis.

Furthermore, in this meta-analysis, we analyzed the diagnostic efficacy of each single diagnostic criterion. Size or shape based criterion was frequently used in assessment of lymph node evaluation by CT and MRI. In general, as the diameters of short axis or long axis adopted as positive criteria increase, the SEN decreased as the SPE increase. Therefore, when short axis diameter was adopted as a positive criterion in MRI examination, we recommended a diameter larger than 8 mm as the best criteria. When long axis criterion was adopted, we recommended a diameter of 12 mm as the cut-off point. In addition, shape based criteria were also used in discriminating metastatic lymph node from benign disease. Based on the evidence provided by this meta-analysis, the lobulated shape should be preferentially adopted. When CT was used in metastatic lymph node detection, short axis diameter >8 mm or long axis diameter >9-10 mm was considered. Besides, if necrosis was found in the lymph node, the diagnostic specificity was greatly improved [102]. Criteria used in DWI were also analyzed. Minimum ADC < 0.0008 was better than mean ADC or relative ADC with a sensitivity of 100% and specificity between 98% and 99%. As regards PET, 5-point grading system is recommended. Moreover, SUV>2 combined with short axis>10 mm is suggested as positive criterion when PET-CT is adopted.

The main results in this meta-analysis were mainly based on patient or region as analytical unit, and node-based data was not mainly applied. The number of studies using node-based data is much smaller than that using patient/region based data. To a surgeon, the most important issue is to learn whether a certain region in pelvic was invaded by malignant lymph node rather than exactly which lymph node was contaminated. Because in clinical setting, if a malignant lymph node was distinguished by imaging technology preoperatively, all the lymph nodes in the malignant lymph node containing region were considered to be excised. In other words, patient/region based data is of more clinical importance than node-based data. In our study, we combined patient-based data with region-based data together. In clinical practice, when one lymph node in a certain pelvic region is malignant, the patient is considered affected by pelvic lymph node malignancy. Once patient is proved to have pelvic lymph node metastasis, the surgical procedure and the prognosis prediction will be similar. This is why we combine region-based data with patient-based data in the current study.

Alongside the listed results, some limitations are present in this systematic review. First, limited data on the diagnostic efficacy of a single criterion were retrieved, thus influencing data pooling with a single criterion. We call for that future studies could focus on this issue and more studies exploring diagnostic efficacy of single criterion are required for further exploration of a high diagnostic efficient criterion. Second, some included studies enrolled a small number of patients, which influenced the accuracy of the results. Third, some studies had a high risk of bias and application concern. This should be improved by further conducting of high quality studies in the future. Last but not least, although we have investigated the potential resource of heterogeneity in study-level factors, the influence of other confounding factors such as skill of medical imaging technologists and pathologists, influence of node dissection for pathological examination, quality of imaging machines could not be considered. In the current meta-analysis, the imaging analysis in the majority of including studies were performed by two imaging technologists respectively which may partially offset the heterogeneity derived by judgment difference of imaging technologists. And for the other factors, they are the problems that all diagnostic accuracy systematic reviews meet. Theories should be raised to solve such problems in the future.

In conclusion, our present study demonstrated that DWI, PET, PET-CT were the top-priority consideration as imaging modalities used for detecting metastatic pelvic lymph node in gynecological carcinoma. DWI is recommended as the first choice for metastasis exclusion and all the other imaging techniques, including CT and MRI, were suitable for metastasis conformation. However, for an early stage lymph node malignancy evaluation, PET or PET-CT might be the better choice. More studies exploring the diagnostic efficacy of detailed criteria are required in the future.

## MATERIALS AND METHODS

According to the protocol set in advance, two reviewers (Gong Y, Wang Q) conducted the study inclusion, data extraction, and risk of bias assessment in duplicate. Disagreements between the two reviews were solved by discussion.

### Inclusion criteria

Inclusion criteria were established as follows: (1) Types of studies: diagnostic test accuracy studies designed as cohort studies; (2) participants: diagnosed as gynecological cancer including uterine cervical cancer, corpus cancer, ovarian cancer, endometrial carcinoma. All these diagnoses should be confirmed by biopsy or pathology; (3) index tests: all kinds of imaging techniques including MRI, DWI, CT, PET, PET-CT, US; (4) reference standard: pathological diagnosis; and (5) targeting conditions: metastasis of the tumor to the pelvic lymph nodes; (5) outcomes: true positive (TP), false positive (FP), false negative (FN), and true negative (TN) (Other outcome variables such as SEN, SPE, +LR and -LR were also considered as they could help to calculate TP, FP, FN and TN).

### Search strategy and study inclusion

Both electronic search and hand-searching were performed for this systematic review.

Bibliographic databases used included MEDLINE (via PubMed, 1946 to March 15th, 2016), EMBASE (via OVID, 1980 to March 15th, 2016), and China National Knowledge Infrastructure (CNKI, 1994 to 1948 to March 15th, 2016). We also performed grey literature searching including Science Paper Online (to 1948 to March 15th, 2016), System for Information on Grey Literature in Europe (OpenSIGLE, 1980 to 2005), and WHO International Clinical Trials Registry Platform (WHO ICTRP, to 1948 to March 15th, 2016). The search strategy of the above databases was designed according to Cochrane Handbook for Diagnostic Accuracy Reviews, draft version 0.4, with a combination of MeSH terms and free text words [103]. The MeSH terms used included: “Pelvis”, “Neoplasms”, “Lymph Nodes”, and “Sensitivity and Specificity”.

We also hand-searched the reference list of all the included studies to retrieve any eligible study missed during the electronic searches.

The two reviewers first scanned the search records (titles and abstracts) and find any potential eligible study. All the recognized records were combined and the full texts of these studies were retrieved. The reviewers further evaluated the full texts and made a final judgment according to the inclusion criteria.

### Risk of bias and applicability assessment

We assessed the risk of bias and applicability using QUADAS-2 and recorded them using Revman 5.3 (Copenhagen, The Nordic Cochrane Centre, The Cochrane Collaboration) [104]. Four domains were included in the assessment tool, such as patient selection, index test, reference standard, and flow and timing. Each domain was assessed in terms of risk of bias, with the first three domains were additionally assessed in terms of concerns regarding applicability. We answered signaling questions provided by QUADAS-2 to evaluate the risk of bias. We tailored the signaling questions to form review-specific guidance as we have done in our previous systematic review assessing cervical node metastasis of head and neck cancer [105].

The signaling questions for this review included:

Patient selection:

Patients enrolled in this study were obtained by a consecutive or random sampling?

Was a case-control design avoided?

Did the study avoid inappropriate exclusions?

Index test:

Were the index test results interpreted without knowledge of the results of the reference standard?

Reference standard:

Was the reference standard likely to correctly classify the target condition?

Were the reference standard results interpreted without knowledge of the results of the index test?

Flow and timing:

Was there an appropriate interval between index tests and reference standard?

Did all patients receive a reference standard?

Were all patients included in the analysis?

The risk of bias for each domain was assessed as high, unclear or low risk of bias. High risk of bias indicated that the answer of at least one signal questions was no; low risk of bias indicated that the answers to all the signal questions in this domain were yes. Unclear risk of bias referred to any situations other than high or low risk of bias. Applicability was also classified as high, unclear or low applicability concerns. The assessment criteria of risk of bias for the whole study were similar to that used in domain evaluation. For all the four domains in a whole study, if the risk of bias for any domains was high, the risk of bias of the whole study would be assessed as high; On the contrary, if all domains had low risk of bias, the risk of bias of the whole study would be assessed as low; any situations other than high or low risk of bias would be assessed as unclear.

### Data extraction

We developed a formal data extraction form for a diagnostic accuracy of our systematic reviews [105, 106], which was used in our previous reviews and pilot-tested on 10% of the included studies in this review. The content of the data extraction form included: Re-evaluation of eligibility; basic information of the study (authors, title, publication time); characteristics of the participants (age, gender, inclusion criteria, tumor types or location, number of included patients); study location (country, patients source); index test and reference standard (details of different imaging techniques and pathological diagnosis, diagnostic criteria, blinding, and consistency of the radiologists); study design (study types and duration); and outcomes (TP, FP, FN, and TN, or any related parameters useful to calculated these outcomes).

### Meta-analysis

We mainly used Stata 14.0 (Stata Corp, College Station, TX, USA) to perform meta-analysis. Statistical heterogeneity was assessed first to help with effect-model chosen in the analysis. Studies with significant clinical heterogeneity were not pooled. In case of a significant clinical heterogeneity, studies were assessed by meta-regression providing evidences for subgroup analysis. We did not assess the reported bias because it was not formally accepted in diagnostic accuracy systematic reviews [107].

### Statistical heterogeneity

The statistical heterogeneity was assessed by Chi^2^ test with the I^2^ statistic. A slight statistical heterogeneity was identified when I^2^≥0.10, and fixed-effect model was used for meta-analysis. When I^2^ < 0.10, significant statistical heterogeneity was identified and random-effect model was used.

### Meta-regression

Log diagnostic odds ratio (logDOR) was considered as the dependent variable of meta-regression. The assignment for each covariate is shown in Appendix Table 1. Multi-covariates meta-regression was performed for each meta-analysis with more than 10 studies included with *P* < 0.10 as statistical significance. Clinical and methodological heterogeneities with potential effects to affect results would undergo subgroup analysis.

### Meta-analysis

The size effect used in the meta-analysis included SEN, SPE, +LR, -LR, DOR, and 95% CIs. We also draw the SROC curve; AUC and Q* (the point of SROC on which sensitivity was equal to specificity) were calculated to reflect synthesized diagnostic accuracy.

### Pair-wise comparison

When needed, pair-wise comparison was performed using z test, which could detect diagnostic differences between SEN, SPE, AUC and Q*. The following formula was used: Z = (VAL1-VAL2)/SQRT(SE12+SE22). VAL indicated the means of SEN, SPE, AUC or Q* and SE was the standard error of the corresponding variables. A value of *P* < 0.05 was considered statistically significant.

## SUPPLEMENTARY MATERIAL FIGURES AND TABLES


