# Supplementary material for: Different imaging techniques for the detection of pelvic lymph nodes metastasis from gynecological malignancies: a systematic review and meta-analysis
Source: Oncotarget. 2016 Oct 27;8(8):14107–25. doi: 10.18632/oncotarget.12959 (PMC5355166; doi:10.18632/oncotarget.12959)
Supplement: Supplementary file 1 [file oncotarget-08-14107-s001.pdf]

Different imaging techniques for the detection of pelvic lymph nodes metastasis from gynecological malignancies: a systematic review and meta-analysis

Supplementary Material

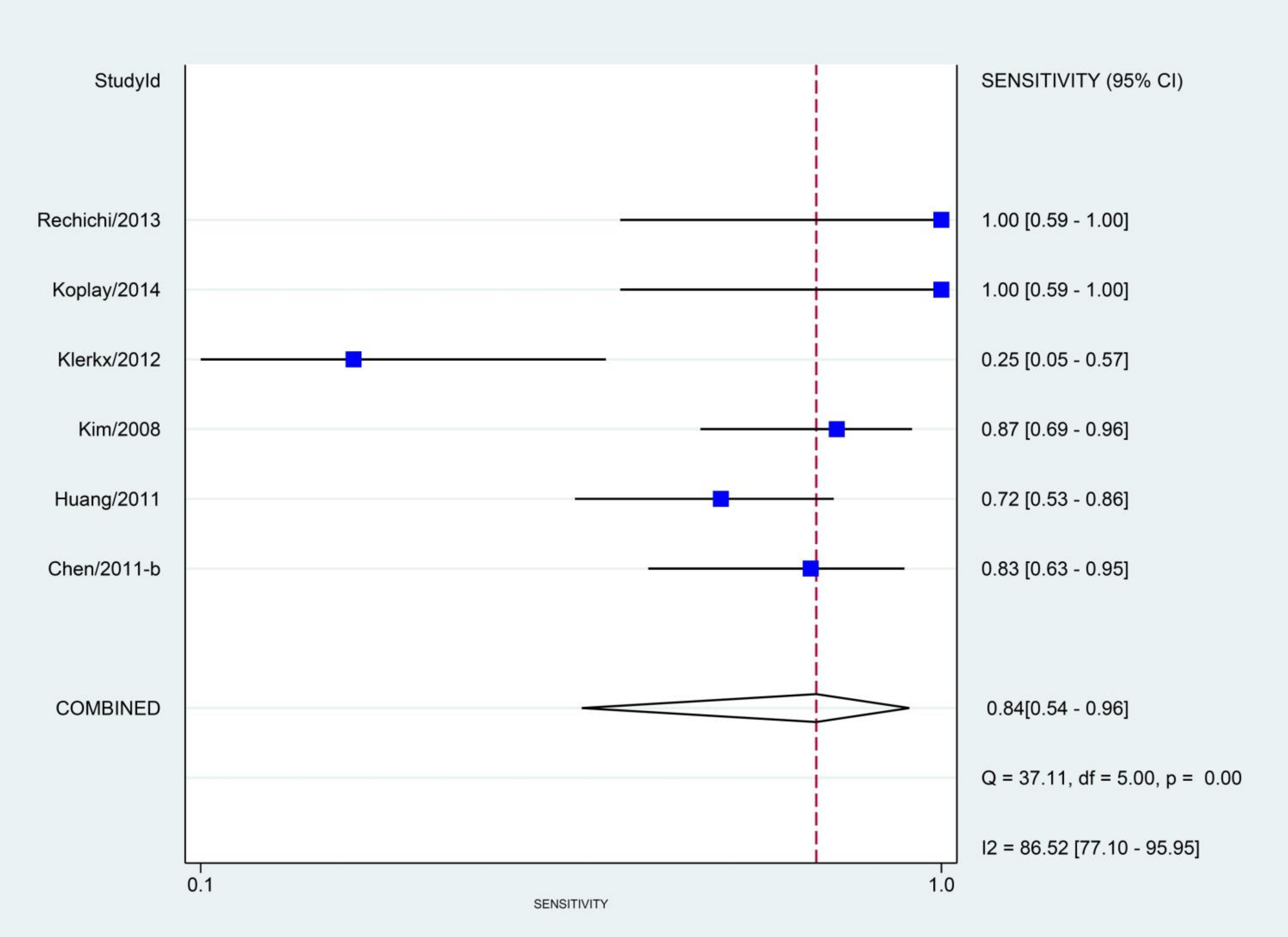

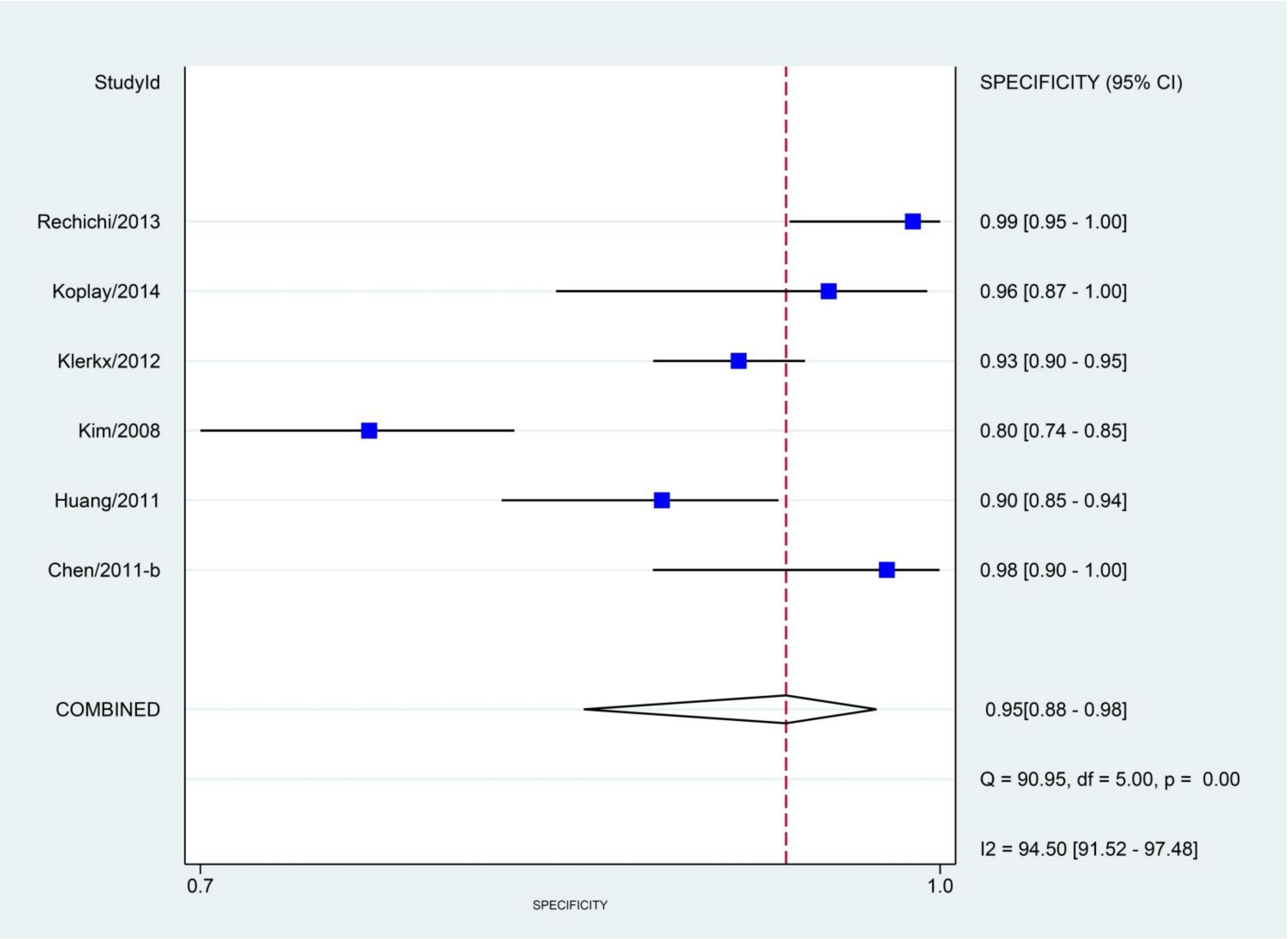
StudyId
SPECIFICITY (95% CI)
Rechichi/2013
0.99 [0.95 - 1.00]
Koplay/2014
0.96 [0.87 - 1.00]
Klerkx/2012
0.93 [0.90 - 0.95]
Kim/2008
0.80 [0.74 - 0.85]
Huang/2011
0.90 [0.85 - 0.94]
Chen/2011-b
0.98 [0.90 - 1.00]
COMBINED
0.95[0.88 - 0.98]
Q = 90.95, df = 5.00, p = 0.00
I2 = 94.50 [91.52 - 97.48]
0.7
1.0
SPECIFICITY

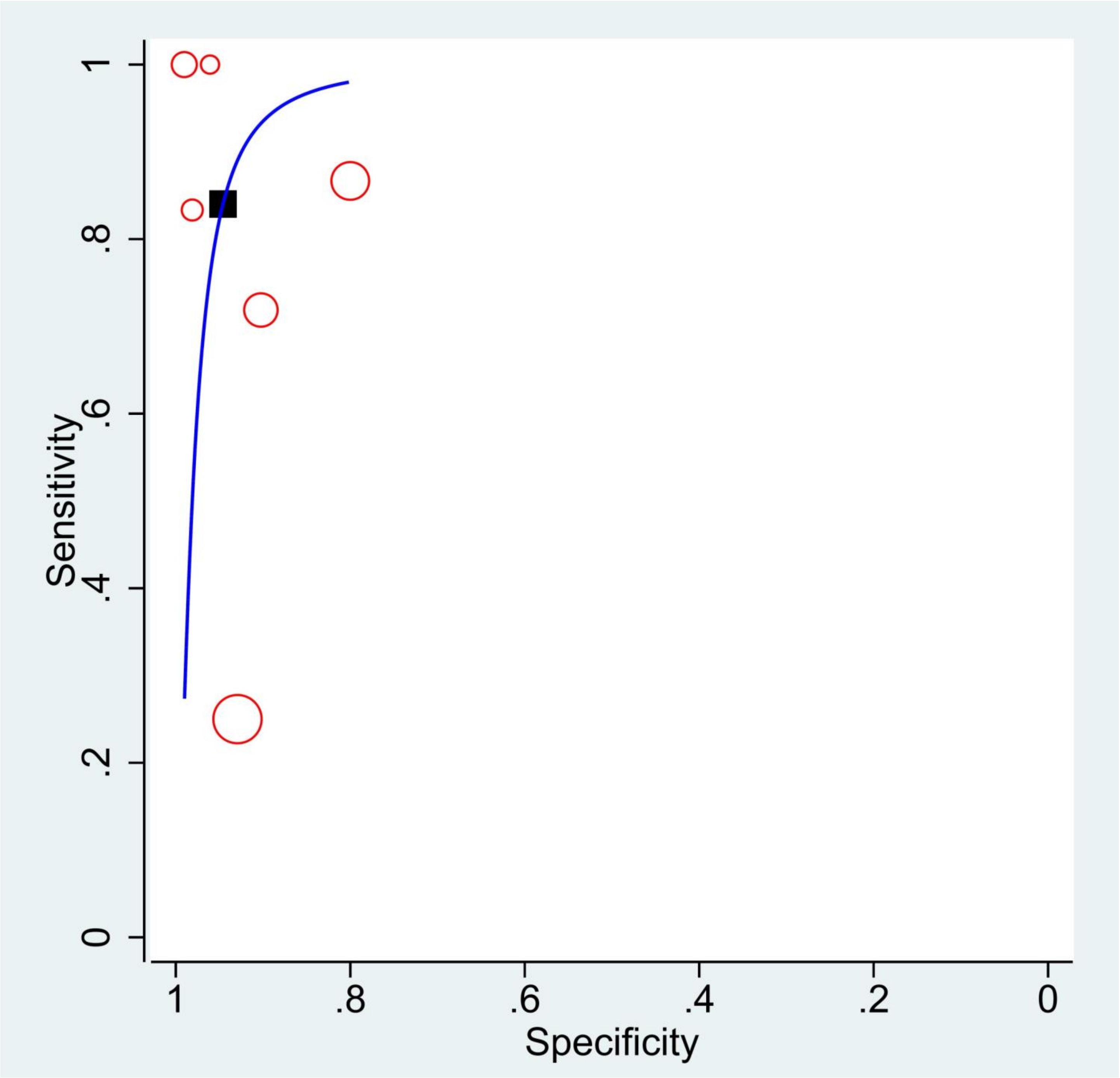

Appendix Figure 1 Results of Meta-analysis Assessing Diagnostic Efficacy of DWI

A. Pooled SEN; B. Pooled SPE; C. SORC curve.

Every circle on the SROC curve represents the coordinate of SEN and SPE in a single study. And the black square represents the summary point where Q* locates.

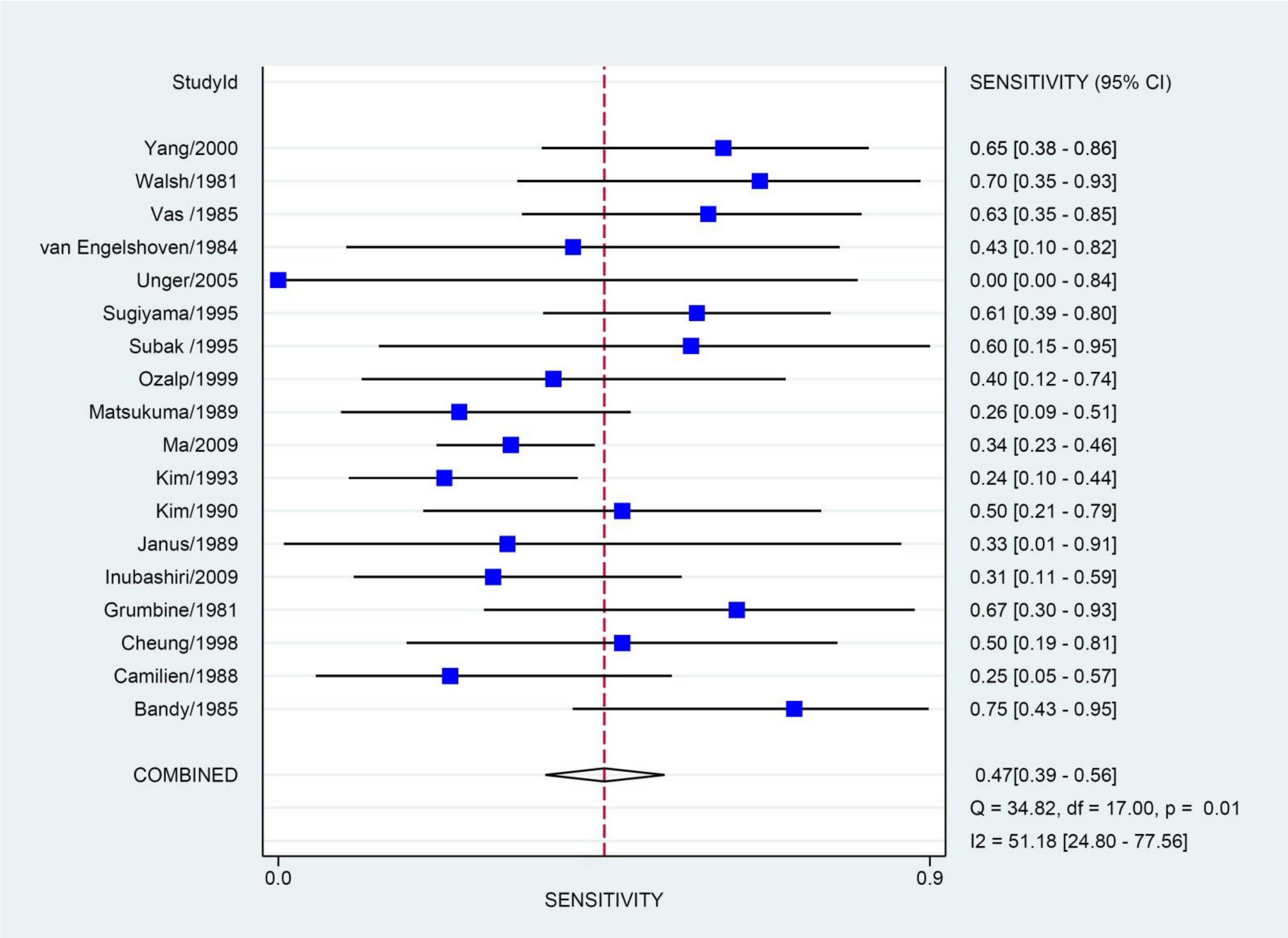
StudyId
SENSITIVITY (95% CI)
Yang/2000
0.65 [0.38 - 0.86]
Walsh/1981
0.70 [0.35 - 0.93]
Vas /1985
0.63 [0.35 - 0.85]
van Engelshoven/1984
0.43 [0.10 - 0.82]
Unger/2005
0.00 [0.00 - 0.84]
Sugiyama/1995
0.61 [0.39 - 0.80]
Subak /1995
0.60 [0.15 - 0.95]
Ozalp/1999
0.40 [0.12 - 0.74]
Matsukuma/1989
0.26 [0.09 - 0.51]
Ma/2009
0.34 [0.23 - 0.46]
Kim/1993
0.24 [0.10 - 0.44]
Kim/1990
0.50 [0.21 - 0.79]
Janus/1989
0.33 [0.01 - 0.91]
Inubashiri/2009
0.31 [0.11 - 0.59]
Grumbine/1981
0.67 [0.30 - 0.93]
Cheung/1998
0.50 [0.19 - 0.81]
Camilien/1988
0.25 [0.05 - 0.57]
Bandy/1985
0.75 [0.43 - 0.95]
COMBINED
0.47[0.39 - 0.56]
Q = 34.82, df = 17.00, p = 0.01
I2 = 51.18 [24.80 - 77.56]
0.0
0.9
SENSITIVITY

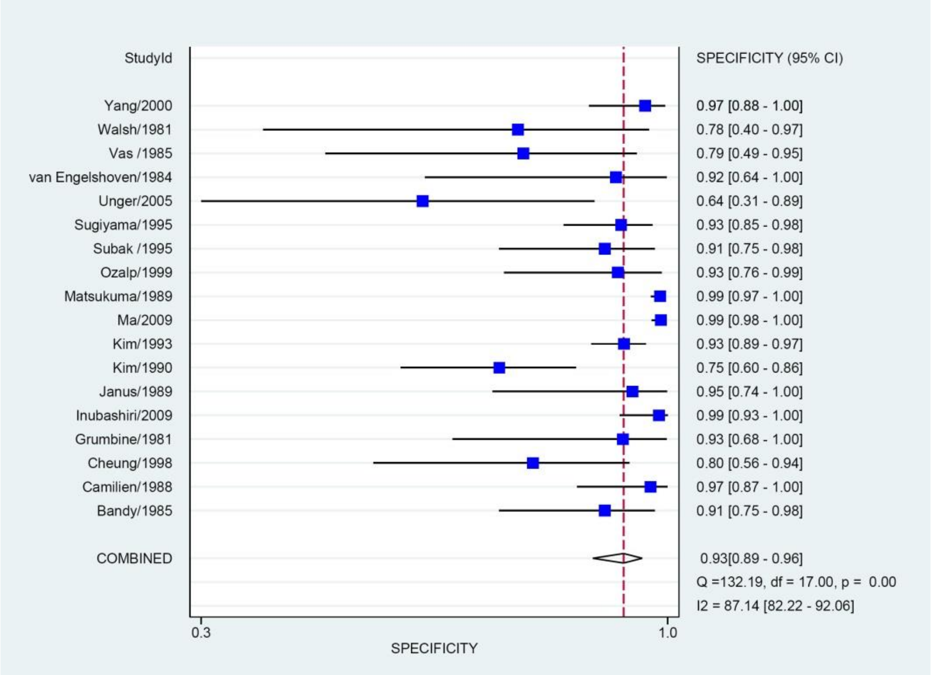
StudyId
SPECIFICITY (95% CI)
Yang/2000
0.97 [0.88 - 1.00]
Walsh/1981
0.78 [0.40 - 0.97]
Vas /1985
0.79 [0.49 - 0.95]
van Engelshoven/1984
0.92 [0.64 - 1.00]
Unger/2005
0.64 [0.31 - 0.89]
Sugiyama/1995
0.93 [0.85 - 0.98]
Subak /1995
0.91 [0.75 - 0.98]
Ozalp/1999
0.93 [0.76 - 0.99]
Matsukuma/1989
0.99 [0.97 - 1.00]
Ma/2009
0.99 [0.98 - 1.00]
Kim/1993
0.93 [0.89 - 0.97]
Kim/1990
0.75 [0.60 - 0.86]
Janus/1989
0.95 [0.74 - 1.00]
Inubashiri/2009
0.99 [0.93 - 1.00]
Grumbine/1981
0.93 [0.68 - 1.00]
Cheung/1998
0.80 [0.56 - 0.94]
Camilien/1988
0.97 [0.87 - 1.00]
Bandy/1985
0.91 [0.75 - 0.98]
COMBINED
0.93[0.89 - 0.96]
Q =132.19, df = 17.00, p = 0.00
I2 = 87.14 [82.22 - 92.06]
0.3
1.0
SPECIFICITY

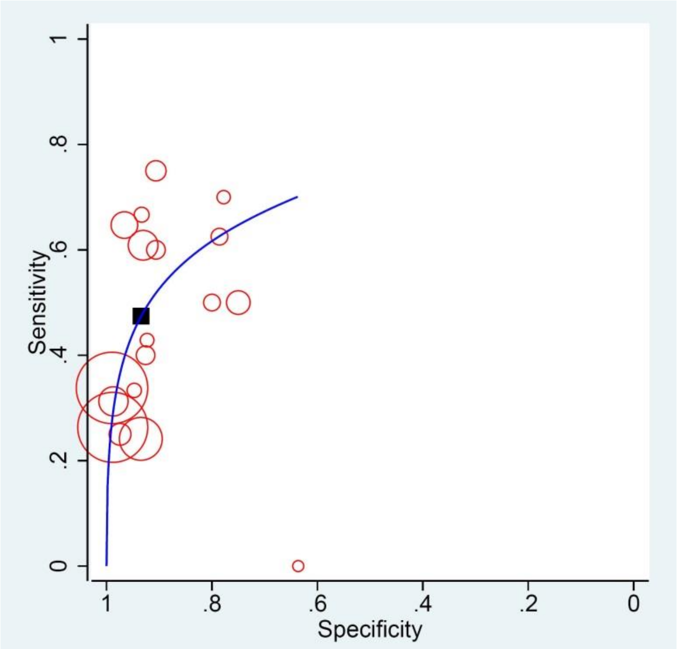

Appendix Figure 2 Results of Meta-analysis Assessing Diagnostic Efficacy of CT

A. Pooled SEN; B. Pooled SPE; C. SORC curve.

Every circle on the SROC curve represents the coordinate of SEN and SPE in a single study. And the black square represents the summary point where Q* locates.

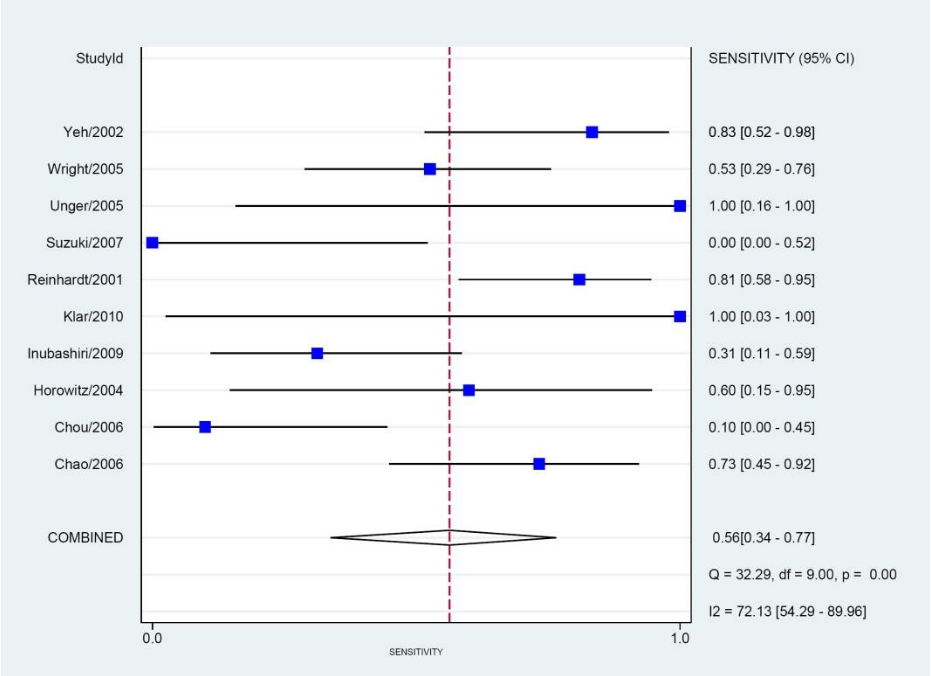

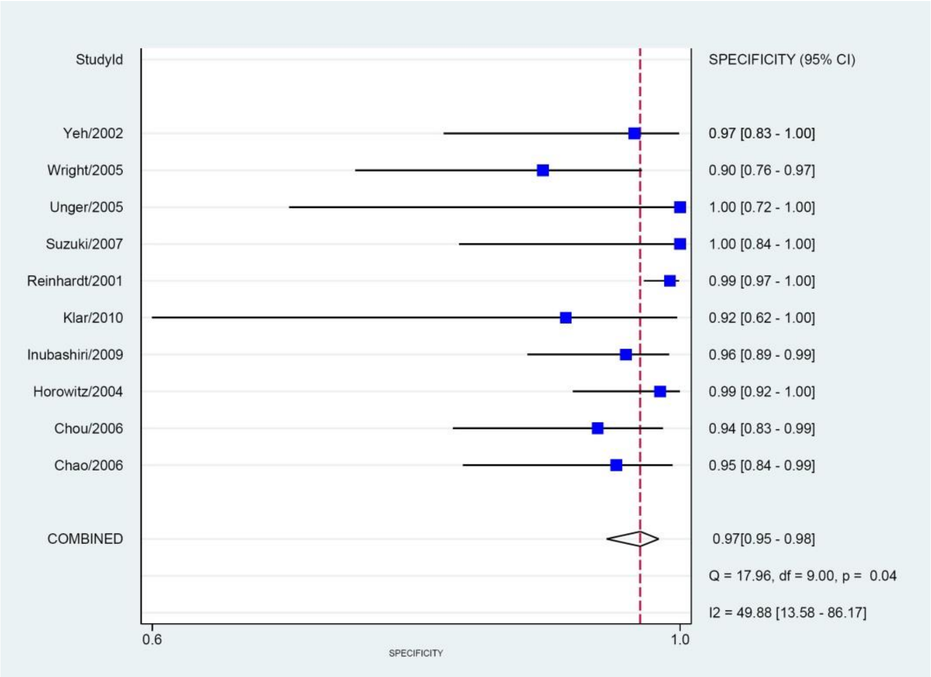

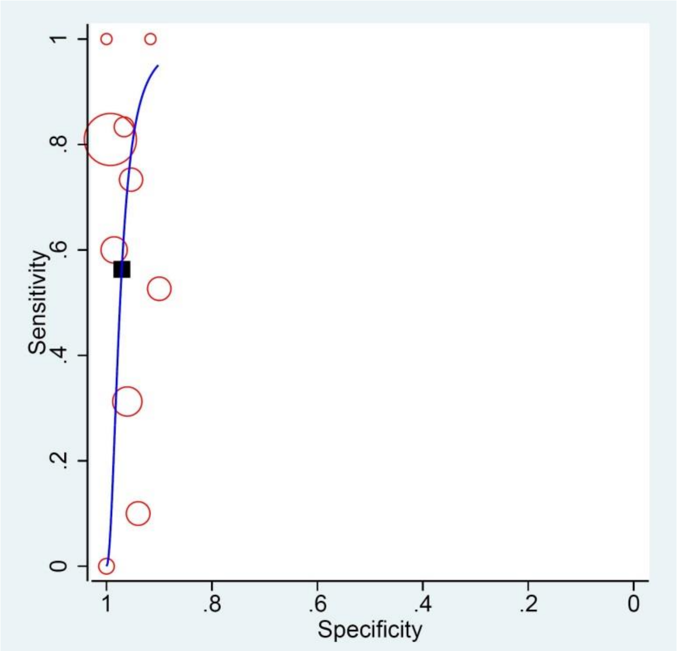

Appendix Figure 3 Results of Meta-analysis Assessing Diagnostic Efficacy of PET

A. Pooled SEN; B. Pooled SPE; C. SORC curve.

Every circle on the SROC curve represents the coordinate of SEN and SPE in a single study. And the black square represents the summary point where Q* locates.

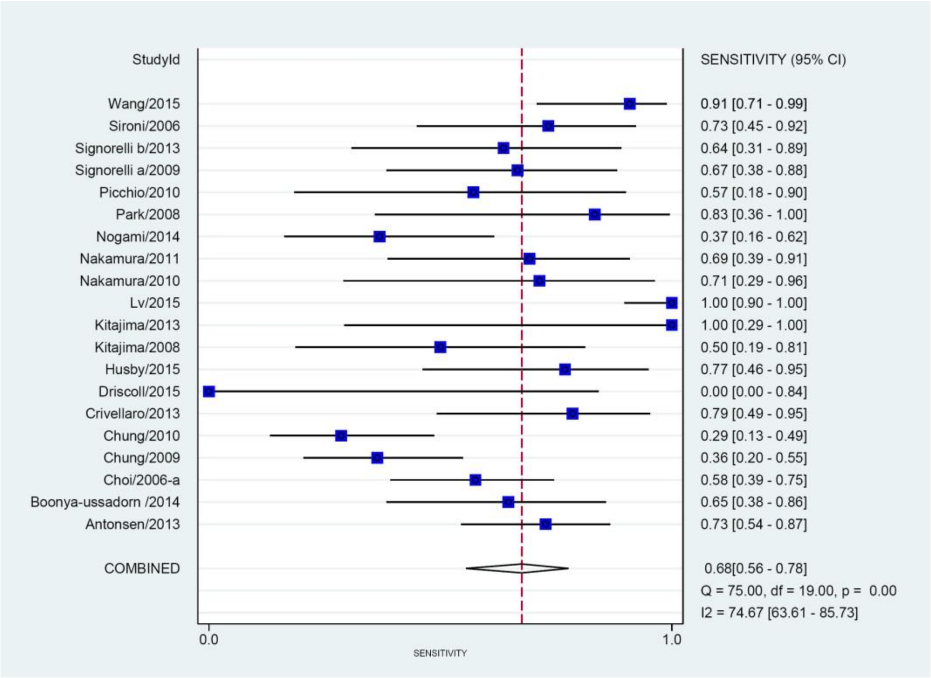
StudyId
SENSITIVITY (95% CI)
Wang/2015
0.91 [0.71 - 0.99]
Sironi/2006
0.73 [0.45 - 0.92]
Signorelli b/2013
0.64 [0.31 - 0.89]
Signorelli a/2009
0.67 [0.38 - 0.88]
Picchio/2010
0.57 [0.18 - 0.90]
Park/2008
0.83 [0.36 - 1.00]
Nogami/2014
0.37 [0.16 - 0.62]
Nakamura/2011
0.69 [0.39 - 0.91]
Nakamura/2010
0.71 [0.29 - 0.96]
Lv/2015
1.00 [0.90 - 1.00]
Kitajima/2013
1.00 [0.29 - 1.00]
Kitajima/2008
0.50 [0.19 - 0.81]
Husby/2015
0.77 [0.46 - 0.95]
Driscoll/2015
0.00 [0.00 - 0.84]
Crivellaro/2013
0.79 [0.49 - 0.95]
Chung/2010
0.29 [0.13 - 0.49]
Chung/2009
0.36 [0.20 - 0.55]
Choi/2006-a
0.58 [0.39 - 0.75]
Boonya-ussadorn /2014
0.65 [0.38 - 0.86]
Antonsen/2013
0.73 [0.54 - 0.87]
COMBINED
0.68[0.56 - 0.78]
Q = 75.00, df = 19.00, p = 0.00
I2 = 74.67 [63.61 - 85.73]
0.0
1.0
SENSITIVITY

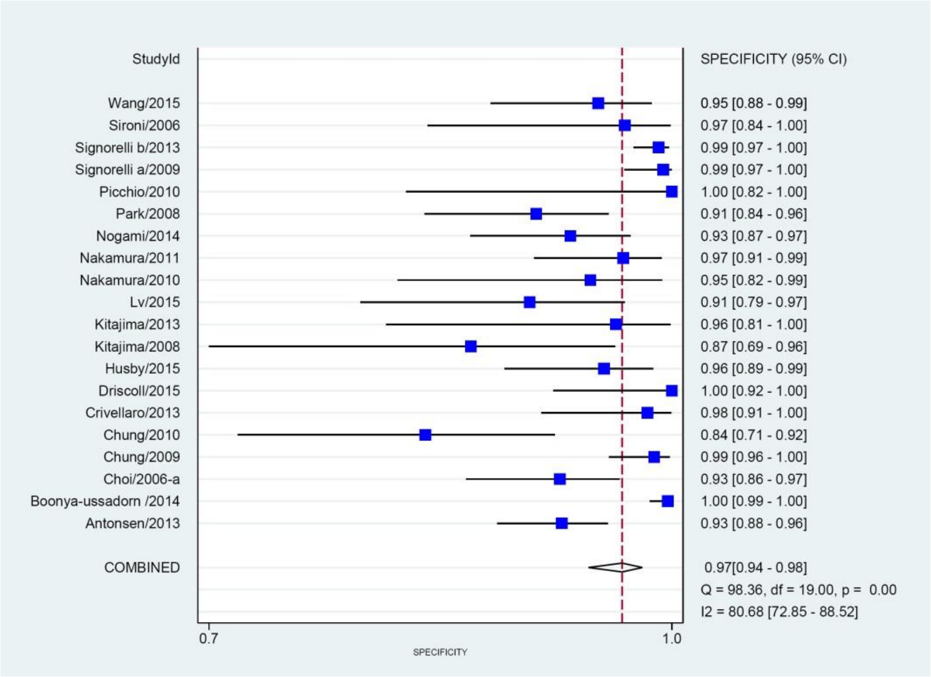
StudyId
SPECIFICITY (95% CI)
Wang/2015
0.95 [0.88 - 0.99]
Sironi/2006
0.97 [0.84 - 1.00]
Signorelli b/2013
0.99 [0.97 - 1.00]
Signorelli a/2009
0.99 [0.97 - 1.00]
Picchio/2010
1.00 [0.82 - 1.00]
Park/2008
0.91 [0.84 - 0.96]
Nogami/2014
0.93 [0.87 - 0.97]
Nakamura/2011
0.97 [0.91 - 0.99]
Nakamura/2010
0.95 [0.82 - 0.99]
Lv/2015
0.91 [0.79 - 0.97]
Kitajima/2013
0.96 [0.81 - 1.00]
Kitajima/2008
0.87 [0.69 - 0.96]
Husby/2015
0.96 [0.89 - 0.99]
Driscoll/2015
1.00 [0.92 - 1.00]
Crivellaro/2013
0.98 [0.91 - 1.00]
Chung/2010
0.84 [0.71 - 0.92]
Chung/2009
0.99 [0.96 - 1.00]
Choi/2006-a
0.93 [0.86 - 0.97]
Boonya-ussadorn /2014
1.00 [0.99 - 1.00]
Antonsen/2013
0.93 [0.88 - 0.96]
COMBINED
0.97[0.94 - 0.98]
Q = 98.36, df = 19.00, p = 0.00
I2 = 80.68 [72.85 - 88.52]
0.7
1.0
SPECIFICITY

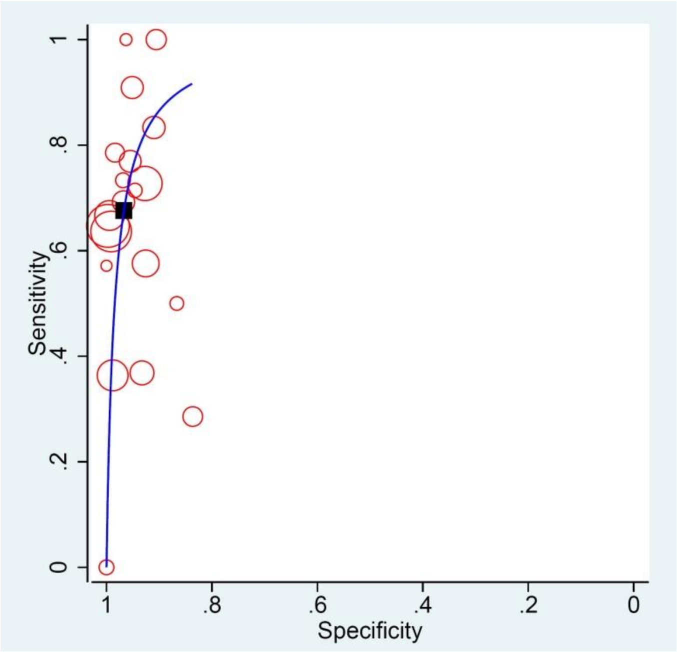

Appendix Figure 4 Results of Meta-analysis Assessing Diagnostic Efficacy of PET-CT

A. Pooled SEN; B. Pooled SPE; C. SORC curve.

Every circle on the SROC curve represents the coordinate of SEN and SPE in a single study. And the black square represents the summary point where Q* locates.

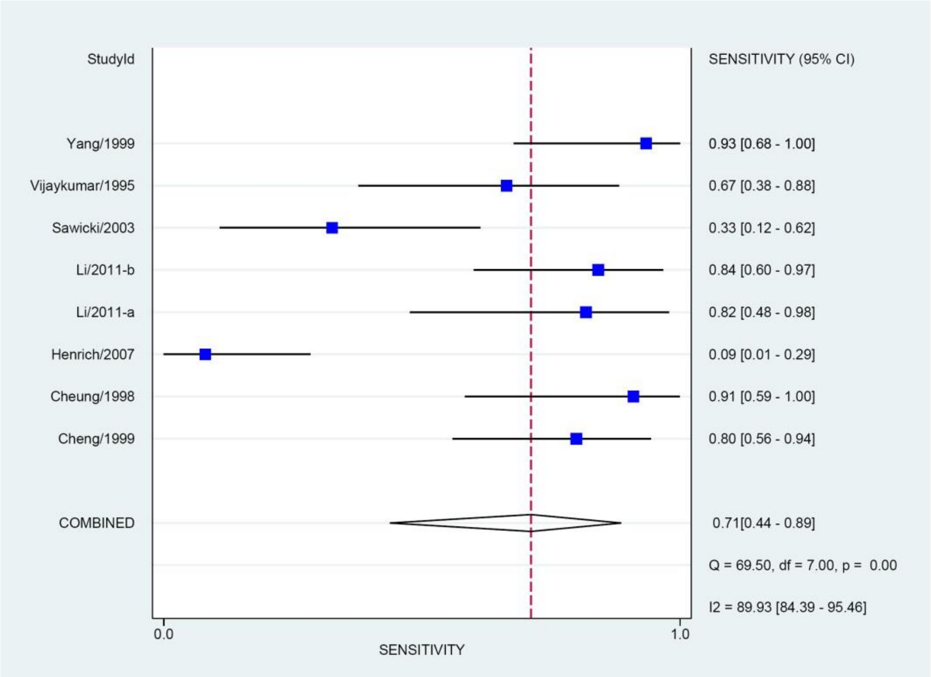

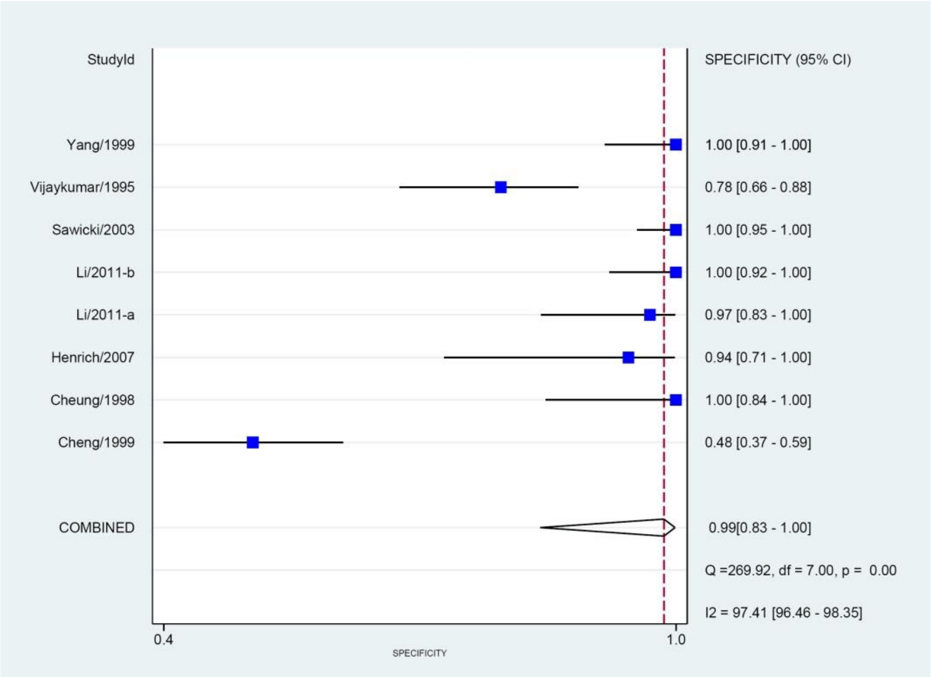

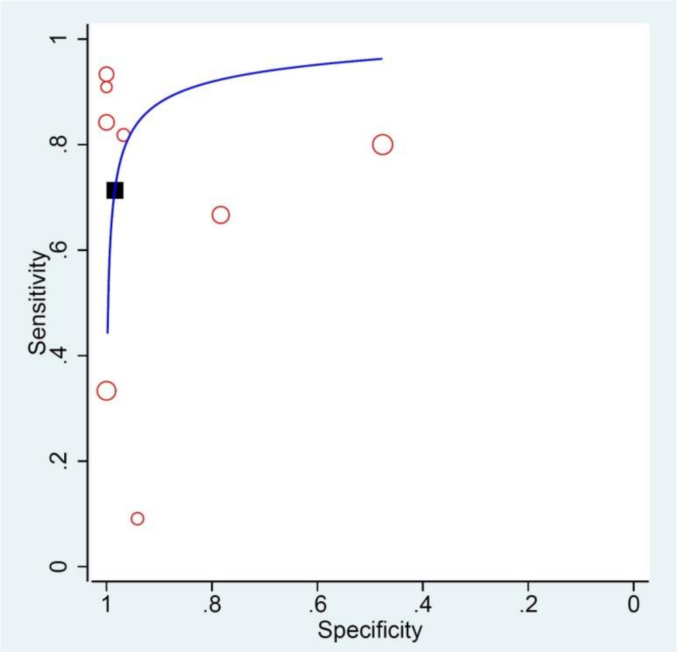

Appendix Figure 5 Results of Meta-analysis Assessing Diagnostic Efficacy of US

A. Pooled SEN; B. Pooled SPE; C. SORC curve.

Every circle on the SROC curve represents the coordinate of SEN and SPE in a single study. And the black square represents the summary point where Q* locates.

Appendix Table 1 Covariate Assignment in Meta-Regression

Covariate	Criteria	Assignment
Publication year	<2010	0
	⩾2010	1
Types of studies	retrospective	0
	prospective	1
Slide thickness	unclear or ⩽4mm	0
	>4mm	1
Intersection gap	unclear or ⩽1mm	0
	>1mm	1
Enhanced	without or partially	0
	with	1
Combined with DWI/CT	without	0
	with	1
Blind of radiologist or pathologist	unclear or unblind	0
	blind	1
Risk of bias	unclear riskor high risk	0
	low risk	1

Appendix Table 2 Meta-regression of MRI on SEN

Parameter	Estimate(95%CI)	Coef	Z	P>|z|
Publication year	0.59 [0.45-0.72]	0.36	0.51	0.61
Types of studies	0.56 [0.42-0.71]	0.26	0.06	0.96
Slide thickness	0.60 [0.48-0.71]	0.40	0.91	0.36
Intersection gap	0.53 [0.35-0.70]	0.11	-0.42	0.67
Enhanced	0.57 [0.41-0.71]	0.26	0.05	0.96
Combined with DWI	0.78 [0.62-0.88]	1.25	2.92	0.00
Blind of radiologist	0.55 [0.45-0.64]	0.18	-0.63	0.53
Blind of pathologist	0.49 [0.34-0.63]	-0.06	-1.20	0.23

Estimate: SEN adjust for other factors.

Appendix Table 3 Meta-regression of MRI on SPE

Parameter	Estimate(95%CI)	Coef	Z	P>|z|
Publication year	0.95 [0.91-0.98]	3.03	0.67	0.50
Types of studies	0.96 [0.91-0.98]	3.06	0.73	0.46
Slide thickness	0.94 [0.90-0.96]	2.74	-0.58	0.56
Intersection gap	0.96 [0.90-0.98]	3.13	0.69	0.49
Enhanced	0.96 [0.93-0.98]	3.27	1.45	0.15
Combined with DWI	0.94 [0.87-0.98]	2.81	-0.11	0.92
Blind of radiologist	0.94 [0.91-0.96]	2.78	-0.68	0.50
Blind of pathologist	0.96 [0.92-0.98]	3.14	1.00	0.32

Estimate: SPE adjust for other factors.

Appendix Table 4 Summary of Meta-analysis (Node as Unit of Analysis)

No	SEN	SPE	+LR	-LR	DOR	AUC	Q*

MR	8	0.52[0.32,0.71]	0.95[0.82,0.99]	11.16[3.61,34.52]	0.51[0.35,0.73]	21.98[8.40,57.51]	0.8118(0.0559)	0.7519(0.0468)
DWI	5	0.88[0.84,0.92]	0.80[0.77,0.82]	5.40[3.21,9.09]	0.14[0.08,0.26]	45.19[14.31,142.69]	0.9373(0.0556)	0.8949(0.0567)
CT	1	0.27	1.00					
PET	1	0.46	0.91					
PET-CT	4	0.47[0.15,0.82]	0.99[0.97,0.99]	368.85[36.13,3765.47]	0.53[0.25,1.13]	694.48[107.36,4492.33]	0.9265(0.0613)	0.8796(0.0559)
US	1	0.69	1.00					

Appendix Table 5 Meta-regression of PET on SEN

**Parameter**	**Estimate(95%CI)**	**Coef**	**Z**	**P>|z|**
Publication year	0.71 [0.57 - 0.83]	0.92	1.39	0.99
Types of studies	0.64 [0.64 - 0.64]	0.59	.	.
Combined with CT	0.68 [0.55 - 0.78]	0.75	1.02	0.31
Blind of radiologist	0.58 [0.44 - 0.71]	0.31	-1.49	0.14
Blind of pathologist	0.66 [0.47 - 0.81]	0.65	0.18	0.86

Estimate: SEN adjust for other factors.

Appendix Table 6 Meta-regression of PET on SPE

Parameter	Estimate(95%CI)	Coef	Z	P>|z|
Publication year	0.97 [0.94 - 0.98]	3.37	-0.16	0.87
Types of studies	0.97 [0.97 - 0.97]	3.55	.	.
Combined with CT	0.97 [0.95 - 0.98]	3.35	-0.42	0.67
Blind of radiologist	0.96 [0.94 - 0.98]	3.25	-0.97	0.33
Blind of pathologist	0.97 [0.94 - 0.98]	3.41	-0.00	1.00

Estimate: SPE adjust for other factors.

Appendix Table 7 Meta-regression of PET on Diagnostic Efficacy

**Parameter**	**I-squared(95%CI)**	**LRTChi**	**P value**
Publication year	0.00 [0.00 - 100.00]	1.91	0.38
Types of studies	100.00[0.00- 100.00]	-0.43	1.00
Combined with CT	0.00 [0.00 - 100.00]	1.22	0.54
Blind of radiologist	35.11 [0.00 - 100.00]	3.08	0.21
Blind of pathologist	0.00 [0.00 - 100.00]	0.03	0.98
